# Effect of a prediction tool and communication skills training on communication of treatment outcomes: a multicenter stepped wedge clinical trial (the SOURCE trial)

**DOI:** 10.1016/j.eclinm.2023.102244

**Published:** 2023-09-25

**Authors:** L.F. van de Water, S.C. Kuijper, I. Henselmans, E.N. van Alphen, E.S. Kooij, M.M. Calff, L.V. Beerepoot, J. Buijsen, W.J. Eshuis, E.D. Geijsen, S.H.C. Havenith, F.F.B.M. Heesakkers, S. Mook, K. Muller, H.C. Post, H. Rütten, M. Slingerland, T. van Voorthuizen, H.W.M. van Laarhoven, E.M.A. Smets

**Affiliations:** a Department of Medical Psychology, Amsterdam UMC Location University of Amsterdam, Meibergdreef 9, Amsterdam, the Netherlands; b Department of Medical Oncology, Amsterdam UMC Location University of Amsterdam, Meibergdreef 9, Amsterdam, the Netherlands; c Amsterdam Public Health, Quality of Care, Amsterdam, the Netherlands; d Cancer Center Amsterdam, Cancer Treatment and Quality of Life, Amsterdam, the Netherlands; e Department of Medical Oncology, Elisabeth-TweeSteden Ziekenhuis, Tilburg, the Netherlands; f Department of Radiation Oncology (MAASTRO), Maastricht University Medical Centre, GROW School for Oncology and Developmental Biology, Maastricht, the Netherlands; g Department of Surgery, Amsterdam UMC Location University of Amsterdam, Meibergdreef 9, Amsterdam, the Netherlands; h Department of Radiation Oncology, Amsterdam UMC Location University of Amsterdam, Meibergdreef 9, Amsterdam, the Netherlands; i Department of Medical Oncology, Flevoziekenhuis, Almere, the Netherlands; j Department of Surgery, Department of Intensive Care Medicine, Catharina Ziekenhuis, Eindhoven, the Netherlands; k Department of Radiation Oncology, University Medical Center Utrecht, Utrecht, the Netherlands; l Department of Radiation Oncology, Radiotherapiegroep, Deventer, the Netherlands; m Department of Radiation Oncology, Radboud University Medical Centre, Nijmegen, Netherlands; n Department of Medical Oncology, Leiden University Medical Center, Leiden, the Netherlands; o Department of Medical Oncology, Rijnstate, Arnhem, the Netherlands

**Keywords:** Prediction model, Communication skills training, Intervention study, Risk communication, Esophageal cancer, Gastric cancer

## Abstract

**Background:**

For cancer patients to effectively engage in decision making, they require comprehensive and understandable information regarding treatment options and their associated outcomes. We developed an online prediction tool and supporting communication skills training to assist healthcare providers (HCPs) in this complex task. This study aims to assess the impact of this combined intervention (prediction tool and training) on the communication practices of HCPs when discussing treatment options.

**Methods:**

We conducted a multicenter intervention trial using a pragmatic stepped wedge design (NCT04232735). Standardized Patient Assessments (simulated consultations) using cases of esophageal and gastric cancer patients, were performed before and after the combined intervention (March 2020 to July 2022). Audio recordings were analyzed using an observational coding scale, rating all utterances of treatment outcome information on the primary outcome–precision of provided outcome information–and on secondary outcomes–such as: personalization, tailoring and use of visualizations. Pre vs. post measurements were compared in order to assess the effect of the intervention.

**Findings:**

31 HCPs of 11 different centers in the Netherlands participated. The tool and training significantly affected the precision of the overall communicated treatment outcome information (*p* = 0.001, median difference 6.93, IQR (−0.32 to 12.44)). In the curative setting, survival information was significantly more precise after the intervention (*p* = 0.029). In the palliative setting, information about side effects was more precise (*p* < 0.001).

**Interpretation:**

A prediction tool and communication skills training for HCPs improves the precision of treatment information on outcomes in simulated consultations. The next step is to examine the effect of such interventions on communication in clinical practice and on patient-reported outcomes.

**Funding:**

Financial support for this study was provided entirely by a grant from the Dutch Cancer Society (UVA 2014-7000).

**Research in context**

**Evidence before this study**

We systematically searched the literature on existing clinical prediction models for treatment of patients with esophageal and gastric cancer. We included several search terms for ‘esophageal cancer’ or ‘gastric cancer’ in combination with search terms for ‘prediction model’, ‘survival’, ‘adverse events’ and ‘quality of life’ to search databases of MEDLINE, EMBASE, PsycINFO, CINAHL, and The Cochrane Library (January, 1st 2000–February 6th, 2017). 47 models were found varying in predicted outcomes, but mostly aimed at survival after curative resection. We were unable to perform meta-analysis due to inadequately reported model calibration and considerable bias in the reported studies. Furthermore, most models lacked external validation, indicating an impediment in applying the models in clinical practice. Moreover, only few models predicted probabilities of side effects or complications and none focused on patient’s health-related quality of life, despite its relevance. We concluded there is a clear need for new prediction models for outcomes of esophageal and gastric cancer and for more investigation on their applicability in clinical practice. To fill this gap, we developed a prediction tool, with underlying validated models, and supporting communication skills training on the use of this tool in clinical practice.

**Added value of this study**

Our current clinical trial shows the effect that clinical application of such prediction models can have on the communication of health care providers about treatment outcomes. Providing health care providers with a tool presenting clear and easy-to-understand visualizations of personalized treatment outcome data, together with training equipping them with the right skills on information giving that is precise and tailored to a patients’ information needs and understanding, affects their information giving in a simulated setting. Application of such an intervention can result in patients receiving information that is more precise, more supported by visualizations, more personalized to clinical characteristics and more tailored to individual patients’ needs.

**Implications of all the available evidence**

We provided the first evidence for the effects of clinically applying prediction models in esophageal and gastric cancer treatment. As we found promising results for the use of the prediction tool in simulated practice, the next step is to investigate the effects on health care providers’ communication in real-life clinical practice. We are currently assessing this effect as part of the same stepped wedge clinical trial (the SOURCE trial), in addition to effects of the combined intervention on patient-reported outcomes, such as their knowledge about the expected treatment outcomes and their evaluation of the decision. If similar effects are found at real-life outpatient clinics, health care providers should be encouraged to implement the tool and training in their daily clinical practice.

## Introduction

Esophageal and gastric cancers are high incidence cancers that cause more than 1.3 million annual deaths worldwide.^1^ An array of treatment options is available both in the curative and palliative setting, comprising different combinations of chemotherapy, radiotherapy and surgery or best supportive care (BSC).^2^^,^^3^ The outcomes of these options differ significantly in terms of survival, risk of side effects and complications, and expected health-related quality of life (HRQoL).^2^^,^^4^^,^^5^ Considering that these pros and cons hold varying importance for each patient, it could be argued that treatment decision making necessitates patients’ individual consideration in a shared decision-making (SDM) process.^6^, ^7^, ^8^ But, irrespective of the specific patient role or decision-making process, it is essential for healthcare providers (HCPs) to thoroughly inform patients about potential outcomes of different treatment options. Existing research indicates that many patients express a desire to receive more information on treatment outcomes.^9^, ^10^, ^11^, ^12^, ^13^ However, currently HCPs underuse clinical outcome data to inform patients on treatment and treatment-related outcomes.^14^^,^^15^

For outcome information to benefit patients, it must be evidence-based, i.e., relying on the best available and most up-to-date evidence. Furthermore, patients desire outcome information to be sufficiently precise, i.e., offering clarity, concreteness, and substantial details.^16^ However, actual treatment outcomes can significantly vary among patients, depending on specific patient characteristics, such as age and performance status, or tumor characteristics, such as number and location of metastases.^17^^,^^18^ Additionally, patients may vary in their personal information needs and preferences dictating the type, amount, and level of detail they wish to receive, and are able to process. Thus, to effectively inform patients, outcome information must not only be evidence-based and precise, but also personalized to clinical characteristics, and tailored to individual patients’ preferences. However, it has been shown that informing patients in such a way is considered challenging by HCPs.^19^, ^20^, ^21^, ^22^

Because to date no personalized and clinically applicable aids exist for HCPs treating patients with esophagogastric cancers,^23^ we iteratively developed an online prediction tool (named ‘Source’), for use in the consultation room.^22^ Additionally, we developed supporting communication skills training (CST) for HCPs to assist them in improving the information that they communicate to patients about treatment outcomes, as CST has previously been proven to be effective in changing oncology HCPs’ communication behaviors.^22^^,^^24^ ‘Source’ is a web-based prediction tool which shows visualizations of personalized data on survival, side effects and complications, and HRQoL, making use of underlying prediction models and meta-analyses.^22^^,^^25^, ^26^, ^27^, ^28^, ^29^, ^30^, ^31^ The blended CST equips HCPs with the ability to effectively convey complex risk and benefit information to patients in a tailored way, using the Source tool during decision making.^22^ Both tool and training underwent pilot testing with promising preliminary evaluation results.^22^ This study aims to investigate the effect of the tool and training on the way HCPs inform patients about treatment outcomes. Primary outcome is the (numerical) precision with which outcome information is given. Secondary outcomes are 1) other characteristics of the communicated outcome information itself, such as the use of visualizations or natural frequencies, 2) communication approaches used by HCPs during the consultation, such as information personalization (to clinical characteristics) and tailoring (to individual preferences), and 3) HCPs’ self-reported satisfaction, intentions and evaluation of the intervention.

## Methods

### Ethics statement

All procedures of the SOURCE trial were judged as needing no further assessment by the institutional medical ethics review boards of AMC (Medical Ethics Review Board AMC; W19_094), VUmc (Medical Ethics Review Board VUmc; 2019.501), UMC Utrecht (Medical Ethics Review Board Utrecht, 20/173) and LUMC (Medical Ethics Review Board Leiden Den Haag Delft; N21.089), and was approved by the local review boards of all study sites. All methods were carried out in accordance with relevant guidelines and regulations. Informed consent was obtained from all subjects.

### Design

**Fig. 1:**
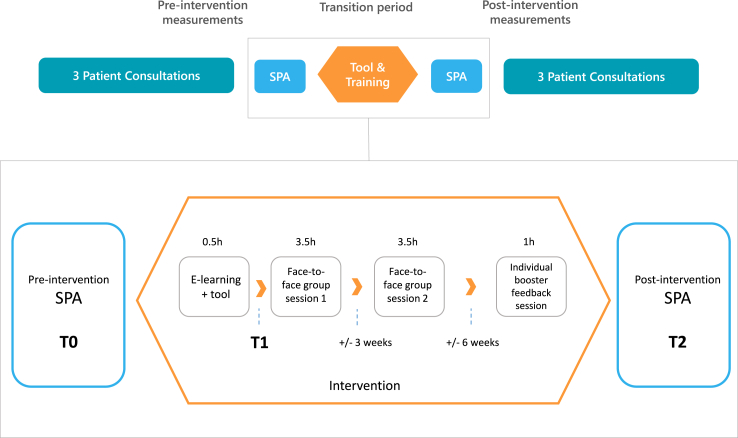
Representation of the study design. Top: simplified design of the SOURCE trial. Bottom: detailed visualization of the current study’s design, including Standardized Patient Assessments (SPAs) and the combined intervention (tool and communication skills training).

This study is part of a multicenter pragmatic stepped-wedge trial (the SOURCE trial, NCT04232735) investigating the combined effect of the online prediction tool and CST on HCPs’ communication of treatment outcomes. The trial examines the effect of the combined intervention in simulated consultations as well as in real life. This paper reports on the effects of the combined intervention in a simulated setting, see Fig. 1 for the design.

Due to the limited number of available subjects within our time window, we had to opt for a design that offers enhanced statistical power compared to a randomized controlled trial. As such, centers were geographically grouped into four parties and the combined intervention was introduced sequentially to each party (non-randomized). As is characteristic of the stepped-wedge design, the intervention was introduced at different moments in time to the four parties, eliminating potential effects in time due to unexpected situations such as the COVID-19 pandemic.

### Setting and participants

Participants were HCPs in surgical, radiation and medical oncology, treating patients with esophageal or gastric cancer and regularly performing treatment information consultations with these patients. A treatment information consultation was defined as a consultation in which one of the HCPs’ main goals is to inform the patient on the outcomes of treatment(s), for example when decisions about treatment have to be made. Oncologists-in-training were also considered eligible, as in the Netherlands they work under supervision yet communicate with patients largely independently.^32^

### Sample size

The SOURCE trial was powered to detect a medium sized effect (Cohen’s d = 0.5) of the combined intervention in real-life consultations, assuming an intracluster correlation (ICC) of 0, and a power of 80%. The intervention was considered successful if a significant difference (α = 0.05) was observed in the precision of information about treatment outcomes provided by HCPs (primary outcome). This resulted in a required sample size of 21 HCPs, i.e., clusters, who would each include real-life consultations with 6 patients (3 pre-intervention measurements, 3 post-intervention measurements). In addition, 2 Standardized Patient Assessments (SPAs) per HCP were conducted (1 pre-intervention, 1 post-intervention). The SPAs were used for current analysis of the effects in a simulated setting, analysis of real-life consultations will be reported on in a separate paper.

### Recruitment

The surgical, radiation and medical oncology departments of academic and nonacademic hospitals were approached through existing networks until at least 30 HCPs were recruited, considering a possible drop out of 30%. HCPs were informed about the study, received an information letter, and were asked for written informed consent.

### Online prediction tool (‘source’)

**Fig. 2:**
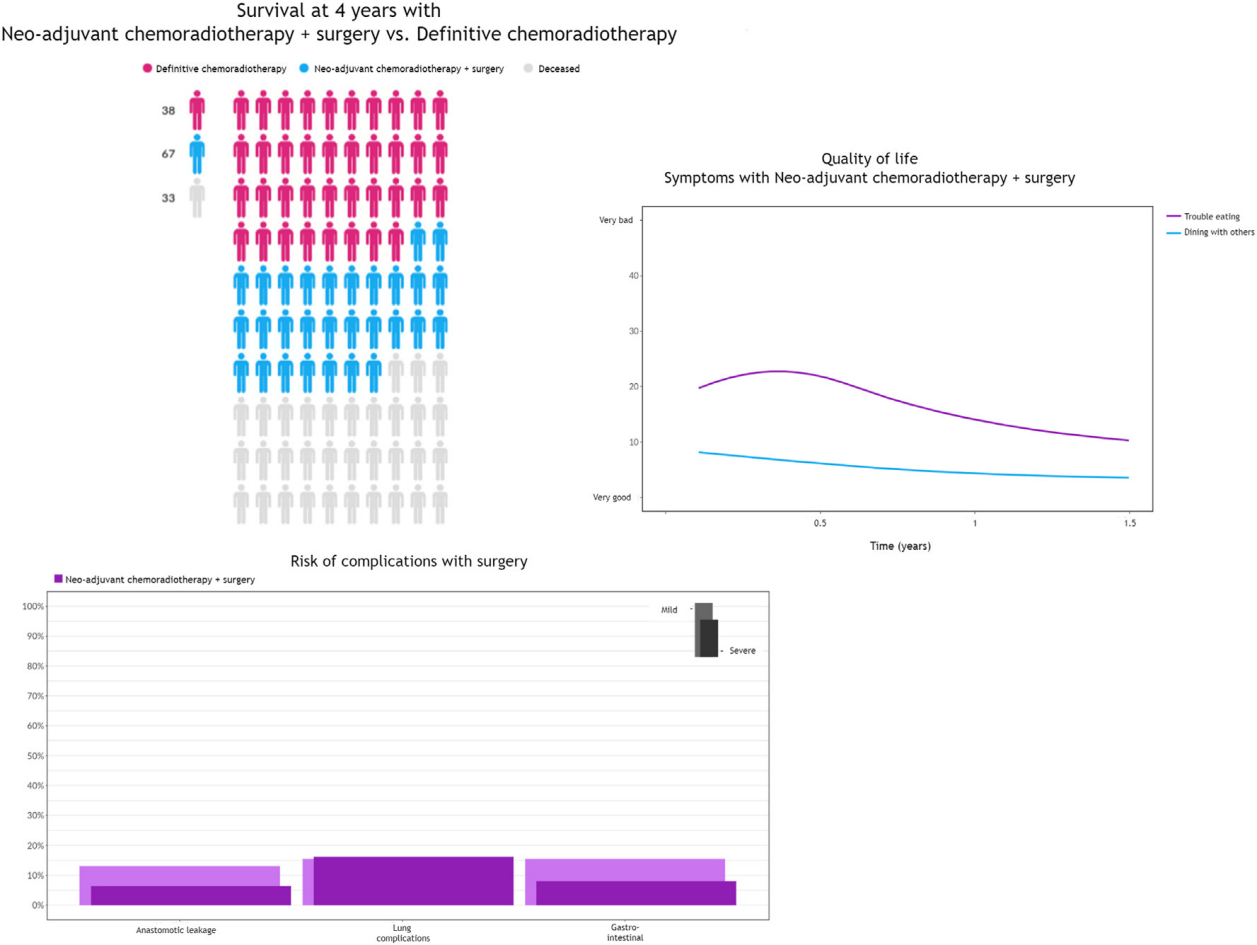
An impression of different visualizations as used for Source (the online prediction tool). For this example a case of curable esophageal cancer was used.

The Source tool contains visualizations of evidence-based, personalized outcome information on chances of survival and surgical complications, and evidence-based information on chances of side effects and expected HRQoL per available treatment option, see Fig. 2. The visualized outcome information is based on self-developed and validated prediction models, meta-analyses and nationwide registry data.^22^^,^^26^, ^27^, ^28^, ^29^, ^30^, ^31^^,^^33^ The tool is designed to be used by HCPs (i.e., physician-assisted) during decision-making consultations. HCPs can tailor the type and amount of information and the types of visualizations to the needs and preferences of an individual patient. Source was developed using an iterative, user-centered approach, involving HCPs as well as patients, patient advocates and field experts.^22^

### Communication skills training (CST)

The blended CST consisted of an e-learning module, two face-to-face group sessions and an individual online booster feedback session, see Fig. 1. Learning goals were for the HCP to 1) be able to name the most important do’s and don’ts for treatment outcome communication (risk and benefit information; knowledge), 2) have a positive outlook on using numbers to inform patients and on their ability to inform patients in an evidence-based, precise, personalized and tailored manner (attitude), 3) be able to use and incorporate the Source tool in their clinical practice (skills) and 4) be able to provide information that is tailored to patients’ informational needs and level of understanding (skills).^22^ HCPs were informed on all functionalities of the tool and its underlying data though the e-learning. The training was developed by a team of experts and experienced trainers (IH, MC, ES, HvL and LvdW) and based on a previous effective training^32^ and review of the literature.^34^, ^35^, ^36^, ^37^ Three out of twenty face-to-face sessions were online, via Zoom, due to COVID-19 regulations. The training was accredited by the Netherlands Association of Internal Medicine, the Dutch Association of Physician Assistants and the Dutch Nurse Specialists Registry.

### Standardized patient assessments (SPAs)

The standardized cases reflected either a scenario of a patient with metastatic gastric cancer opting for palliative treatment (medical oncologists) or of a patient with localized esophageal cancer opting for curative treatment (surgical and radiation oncologists), who met with the HCP to discuss available treatment option(s). Two scripts describing the background of two rather highly educated patients (accountant or archeologist) were attached to both scenarios,^32^ resulting in four different cases (see Appendix 2). HCPs received a simulated medical file. Background stories and actors were counterbalanced, i.e., randomly assigned between pre- and post-intervention for each HCP. Two professional male actors were instructed to play the cases in a standard way, which was not overly emotional,^32^^,^^38^ and not to initiate discussion of treatment outcomes. The patient script included a set of standard questions and a few “if then” rules, including the instructions to ask about survival benefits or risks of side effects only when certain outcomes of treatment were addressed by the HCP. All patient scripts and medical files, based on those of a previous RCT, were further developed in a multidisciplinary team and adjusted based on a pilot study.^22^^,^^32^^,^^38^

SPAs took place in consultation rooms at the hospitals’ outpatient clinics and online, via GoToMeeting, Skype or Zoom, due to COVID-19 restrictions. Whether the consultations occurred in person or online was kept constant between pre- and post-measurements. SPAs were either audio-recorded (in person) or video-recorded (online) and stored safely according to the General Data Protection Regulation (GDPR) and were end-to-end encrypted. Time intervals between the SPA’s and the start of the training period varied as a result of COVID-19 restrictions between 1 and 6 months (T0–T1) and between 4 and 9 months (T1–T2). Total duration of the training period (e-learning–booster feedback session) was ±9 weeks for each HCP.

### Measurements

#### Sample characteristics

Participants: at T0, HCPs reported their gender, age, nationality, function (medical specialist, oncologist-in-training, physician assistant, nurse specialist), expertise (surgical, radiation or medical oncology), years of experience (including residency) and receipt of communication skills training during medical school and/or residency (yes/no).

SPAs: HCPs rated the SPA’s perceived realism and comparability to clinical practice on a Visual Analogue Scale (VAS), at both pre- and post-intervention SPAs (T0, T2).

#### Primary outcome

Observed (numerical) precision of communicated outcome information was considered the primary outcome. As a validated measure for this outcome was not at hand,^39^ the Outcome Information Scale for OesophagoGastric cancer (OIS-OG) was developed. For this scale, treatment outcome information was grouped in four distinct outcomes categories: A) survival, B) side effects and complications, C) HRQoL, and D) treatment response or recurrence. These outcome categories were further broken down into information about individual items of different treatments and complaints, such as ‘chemotherapy’ or ‘nausea’, together forming a coding framework. Every utterance by the HCPs regarding outcome information was analyzed and assessed within the coding framework to evaluate the numerical precision. This assessment aimed to determine the level of richness and detail with which the information was conveyed by the HCPs on a scale from one, e.g., ‘I don’t know’, to four, e.g., ‘50% of people’. For each of the coding framework’s individual items, only the consultations’ maximal score (i.e., most precise utterance) was included in the analysis. All consultations were independently coded by two coders, who were blinded to the experimental condition. For a full description of the OIS and the coding process, see Appendix 1.

#### Secondary outcomes

Both on the level of the SPA, as a whole, as well as on the level of the items that were coded on the primary outcome, we coded several secondary outcomes. See Table 1 for a description of all secondary outcomes.

**Table 1:** Secondary outcomes of the standardized patient assessments (SPAs).

Outcome	Time	Measure	Example
Observations—per SPA			
Number of remarks/attempts to personalize outcomes	T0, T2	Total number of remarks per SPA	*“For patients with your specific tumor, with your age and condition, the chances are..”*
Number of attempts to check current knowledge/understanding	T0, T2	Total number of remarks per SPA	*“What did you understand from the information the gastroenterologist gave you about this treatment?”*
Number of attempts to tailor information to individual needs/preferences	T0, T2	Total number of remarks per SPA	*“Would you like to hear a more general description of the chances of survival or would you like me to give more precise information, in numbers?”*
SPA duration	T0, T2	Total duration, based on video and audio recordings	–
Observations—per item			
Initiative for discussing outcome information	T0, T2 (per item)	Simulated patient (SP)/health care provider (HCP)	Initiative Simulated Patient–*SP: “Doctor, when you say that the chances of survival are better, what difference are we talking about?” HCP: “Well,..”* Initiative Health Care Provider–*HCP: “Let me tell you some more about the difference in chances of survival between these treatments”*
Use of supporting visualization	T0, T2 (per item)	Present/absent	*“as you can see in this graph..”*
Use of time frame	T0, T2 (per item)	Present/absent	*“At 4 years after diagnosis, about 70% of patients is still alive”*
Use of natural frequency	T0, T2 (per item)	Present/absent	*“70 out of 100 patients”*
Use of percentage	T0, T2 (per item)	Present/absent	*“70%”*
Use of framing	T0, T2 (per item)	One/multiple manners (e.g., *positively and negatively or in multiple scenarios*)	Positive + negative framing: *“70 out of 100 patients are alive after 5 years, this means 30 out of 100 have died”* Multiple scenarios: “
Use of uncertainty communication	T0, T2 (per item)	Present/absent	*“these are just chances, but I don’t know what it will be like for you specifically”*
Outcome information about clinical trial treatment	T0, T2 (per item)	Yes/no	*“we can also offer you treatment B if you participate in this clinical trial …”*
HCP questionnaires			
Satisfaction with communication	T0, T2	Patient satisfaction questionnaire—physician version (PSQ-5; 5 items)^11^	*“How satisfied are you with the adequacy of the information you gave to this patient?”*
Clinical behavioral intentions—attitude and intentions towards informing patients using numbers	T0, T2	Subscales of the continuing professional development scale (CPD; 12 items)^40^: *Intention to adopt a behavior; Social influence; Beliefs about capabilities; Moral norm; Beliefs about consequences*	*“In my day-to-day practice I intend to use numbers to inform patients about treatment outcomes” (subscale: Intention to adopt a behavior)*
Evaluation of e-learning + CST	T1, T2	Self-developed evaluation surveys (20 + 12 items)^32^^,^^41^	*“In your opinion, how helpful was the training for your daily clinical practice?”*

### Statistical analyses

#### Primary outcome

For each of two coders, all item scores for precision were summarized per outcome category (survival, side effects and complications, HRQoL, and treatment response or recurrence) and overall (all outcome categories together). The particular items (treatment options, side effects, etc.) and the number of items that were coded as having been discussed by HCPs, differed between HCPs. As such, the scales on which the precision of information provided in the SPAs were scored, differed between HCPs. For example, a HCP who was coded to have discussed three items, each with a 1–4 Likert scale, scored on a theoretical 3–12 scale. To account for these scale differences between HCPs, the mean of the items across outcome categories was calculated and rescaled to a 0–100 scale, taking into account the number of items that were coded for each HCP. The formula for this transformation can be found in Appendix 1. Per SPA, rescaled scores were then averaged over the two coders for use in paired samples analysis of the difference between pre- and post-consultation. Based on the ordinal nature of the data and the assumed non-normal distribution of the observed scores, we used non-parametric paired samples Wilcoxon signed rank tests to test pre- and post-differences (α = 0.05). Considering the educational nature of the intervention, we did not hypothesize lower numerical precision after the intervention a priori, and therefore specifically tested our hypothesis that numerical precision increased significantly, using one-tailed tests for the primary outcome. Analysis was performed using R, version 4.0.3, and R Studio, version 1.3.

In addition to analyses of the total sample, we also performed separate analyses stratified to the curative and the palliative setting. If significant effects on the overall precision of outcome communication in either of the settings were found, further additional analyses were performed for each of the four the outcome categories separately. Effect sizes (r) were calculated by dividing the z-statistic by √N (r = 0.1–0.3 small effect, r = 0.3–0.5 moderate effect, r > 0.5 large effect). Moreover, in the event of significant pre- and post-differences, the initiative taken by the simulated patient to elicit utterances on treatment outcomes was analyzed as an independent covariate to rule out the possibility that an effect of the intervention could be explained by the simulated patient taking more initiative in one of the experimental conditions. This hypothesis was tested by modeling the difference between T0 and T2 (Δ) using the patients’ initiative as a covariate in a proportional odds model, which is a generalization of non-parametric models.^42^ The effect of patients’ initiative was tested for significance, using the RMS package in R. The assumption of proportional odds was formally tested using the Brant test for each proportional odds model that was fitted.

#### Secondary outcomes

Secondary outcomes were analyzed in a similar manner as the primary outcomes, using paired samples Wilcoxon signed rank tests (α = 0.05). Secondary outcomes coded at the item level (e.g., use of supporting visualizations, use of time frames, etc.) were summarized like the primary outcome, but using frequencies instead of scale scores. For each coder, frequencies were divided by the total amount of items scored by this coder to calculate the relative frequency. The relative frequency was then averaged over the two coders. Due to the large number of secondary outcomes, all secondary analyses were accounted for multiple testing using familywise type-I error correction with the Bonferroni method. Familywise correction here implied grouping ‘families’ of tests together that analyzed the same subdivisions of the sample. Bonferroni correction was applied by multiplying by the total number of tests performed per family of tests.

### Role of the funding source

Financial support for this study was provided entirely by a grant from the Dutch Cancer Society (UVA 2014-7000). The funding agreement ensured the authors’ independence in designing the study, interpreting and analyzing the data, writing and publishing the report. LvdW, SK, EvA and EK had full access to all data reported in the manuscript. LvdW, HvL and ES take responsibility for the decision to submit for publication.

## Results

After inviting a total of 56 HCPs, 40 HCPs showed interest in participation and signed informed consent. Of these, 31 HCPs from 11 academic and non-academic centers completed the SOURCE trial (participated in two SPAs and included six patients), including 11 HCPs from surgical departments, 8 from radiation oncology departments and 12 from medical oncology departments. Nine Dropouts occurred during the trial due to a variety of reasons, such as pregnancy, illness, and organizational changes. SPAs took place from March 2020 to July 2022, when all SPAs of 31 participating HCPs were collected. See Table 2 for participant and SPA characteristics.

**Table 2:** Health care provider (HCP) and standardized patient assessment (SPA) characteristics.

HCP characteristics (n = 31)	
Age in years, M (SD)	46.1 (8)
Gender, n (%) male	16 (52)
Nationality, n (%) Dutch	31 (100)
Job position, n (%)	
Medical specialist	27 (87.1)
Oncologist-in-training	2 (6.4)
Physician assistant	1 (3.2)
Nurse specialist	1 (3.2)
Specialty, n (%)	
Medical oncology	12 (38.7)
Radiation oncology	8 (25.8)
Surgical oncology	11 (35.5)
Center employed at, n (%)	
Academic	18 (58.1)
Non-academic	13 (41.9)
Professional experience in years, M (SD)	12 (8)
Communication skills training during, n (%) yes^a^	
Medical school	23 (74.2)
Residency	14 (45.2)

SPA characteristics (n = 62)	Pre/T0 (n = 31)	Post/T2 (n = 31)
Realism/comparability, M (SD)		
Perceived realism (scale 0–10)	7.58 (1.48)	7.42 (1.18)
Perceived comparability (scale 0–10)	7.16 (1.90)	6.61 (1.76)
Location, n (%)		
Online	21 (67.7)	21 (67.7)
Outpatient clinic	10 (32.3)	10 (32.3)
Actor, n (%)		
Actor 1	14 (45.2)	21 (67.7)
Actor 2	17 (54.8)	10 (32.3)
Case, n (%)		
Case 1 (curative or palliative)	14 (45.2)	17 (54.8)
Case 2 (curative or palliative)	17 (54.8)	14 (45.2)

a 3 HCPs indicated ‘non applicable’.

### Primary outcome

**Fig. 3:**
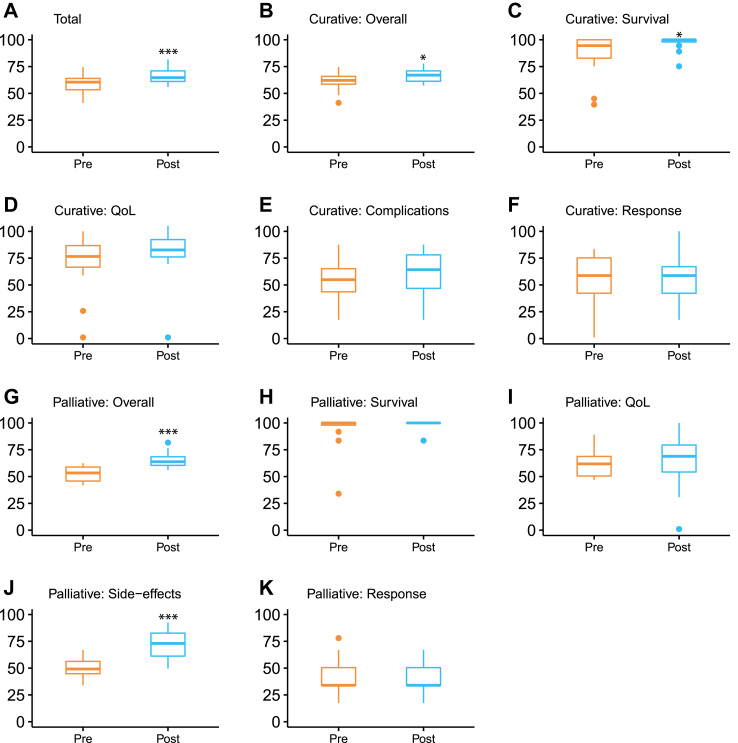
Boxplots displaying spread around the median scale scores (0–100) of the precision of outcome communication for the total sample (A; N = 31), curative cases only (B–D; N = 19) and palliative cases only (F–J; N = 12), for overall outcome communication and all outcome categories separately. ∗*p* ≤ 0.05, ∗∗*p* < 0.01, ∗∗∗*p* < 0.001.

The Source tool and training had a significant positive effect on the precision of the overall communicated treatment outcome information (*p < 0.001; r = 0.63, large effect, median difference 6.93, IQR (−0.32 to 12.44)*). For curative cases, overall treatment outcome information (*p = 0.013; r = 0.51, large effect, median difference 5.27, IQR (−3.91 to 11.84))* and information about survival (*p = 0.029; r = 0.44, medium effect, median difference 0, IQR (0–12.38)*) were significantly more precise after the intervention. We did not find a significant effect on precision regarding information about side effects and complications, HRQoL and treatment response or recurrence. For palliative cases, overall treatment outcome information (*p = 0.001; r = 0.84, large effect, median difference 10.36, IQR (2.98–16.26)*) and information about side effects (*p < 0.001; r = 0.86, large effect, median difference 27.06, IQR (7.91–31.12)*) were significantly more precise after the intervention. Information about survival, HRQoL and treatment response or recurrence was not. See Fig. 3. See Appendix 3 for an overview of which items (treatment options, side effects, etc.) were discussed by HCPs.

Results from the proportional odds models did not indicate that the difference in simulated patient initiative for utterances on treatment outcomes significantly predicted the difference in overall precision between T0 and T2 for both curative and palliative cases across the outcome categories (*p > 0.05 for all*). For curative cases, the assumption of proportional odds was not violated. Due to the small sample size and the relatively large number of ordered categories, the Brant test could not be computed for the models for palliative cases and could therefore not be assessed.

### Secondary outcomes

On the SPA level, the number of remarks indicating that treatment outcomes were personalized to patients’ clinical characteristics was significantly higher after the intervention, for curative cases (*p = 0.001, median difference 2.00, IQR (1.00–3.50)*) as well as palliative cases (*p = 0.032, median difference 1.50, IQR (1.00–2.00)*). Also the number of attempts to tailor information to individual information needs or preferences was significantly higher after the intervention for both curative (*p = 0.001, median difference 2.00, IQR (1.50–4.50)*) and palliative cases (*p = 0.004, median difference 2.00, IQR (1.75–3.00)*). The duration of the consultation was not significantly different between pre- and post-intervention, as were HCPs’ satisfaction, their clinical behavioral intentions to use numbers or their number of attempts to check patients’ current knowledge and understanding. See Table 3 for an overview.

**Table 3:** Results for secondary outcomes coded on the level of the Standardized Patient Assessment (SPA).

Outcome	Median pre/T0	Median post/T2	*p*-value
Curative cases			
Number of remarks/attempts to personalize outcomes	0	3	<0.001^a^
Number of attempts to check current knowledge/understanding	3	5	1.103
Number of attempts to tailor information to individual needs/preferences	1	4	0.001^a^
Palliative cases			
Number of remarks/attempts to personalize outcomes	0	2	0.032^a^
Number of attempts to check current knowledge/understanding	2	3.5	0.136
Number of attempts to tailor information to individual needs/preferences	1.5	4	0.004^a^
Overall (both curative and palliative cases)			
SPA duration (min.)	30	37.5	0.245
HCP satisfaction with communication (1–10)	7.02	7.36	1.416
HCP clinical behavioral intentions (1–7):			
Intention to adopt a behavior	5	5.5	0.438
Social influence	4	4.333	2.554
Beliefs about capabilities	5.333	5.333	1.005
Moral norm	5	5.5	0.108
Beliefs about consequences	5	5	2.497

Note: All *p*-values were familywise corrected for multiple testing using Bonferroni corrections, by multiplying the *p*-values with the total number of tests performed per family of test. As such *p*-values can be larger than one and significance levels remain 0.05.

a Significant at α = 0.05.

**Fig. 4:**
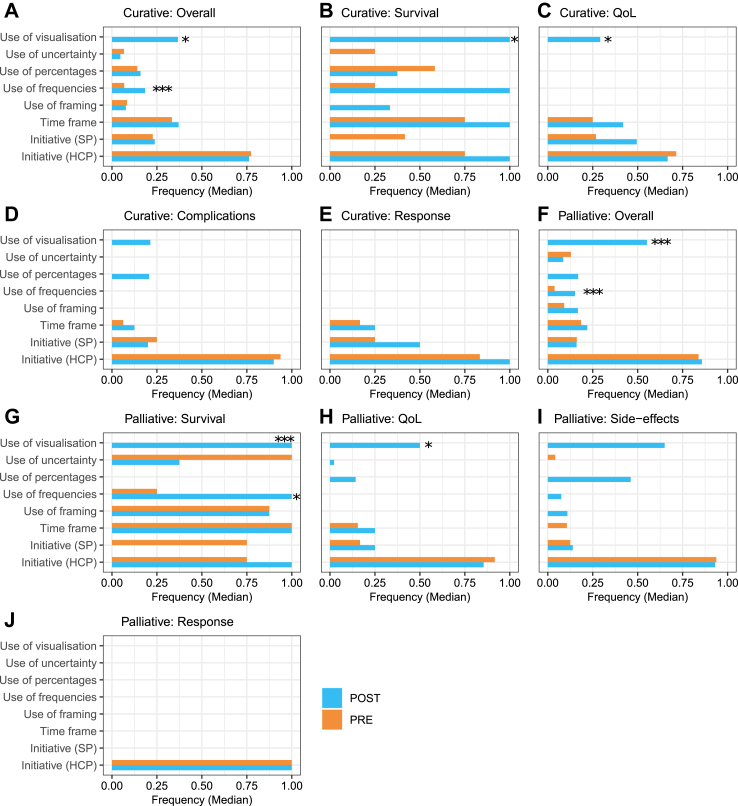
Median frequency of secondary outcomes coded on the item level, corrected for the amount of items communicated in the outcome category (relative frequency). All outcome categories and overall outcome communication are displayed separately, for curative cases (A–E) and palliative cases (F–J). Initiative for the for discussing the outcome information was coded as either coming from the Simulated Patient (SP) or from the Health Care Provider (HCP). ∗*p* ≤ 0.05, ∗∗*p* < 0.01, ∗∗∗*p* < 0.001.

On the item level, the relative number of visualizations used in the consultation was systematically higher after the intervention for almost all outcome categories in the curative and palliative cases, although only significantly higher for overall outcome communication (*cur.: p < 0.001, median difference 0.34, IQR (0.16–0.52), pall.: p = 0.045, median difference 0.51, IQR (0.48–0.56)*), survival information (*cur.: p < 0.001, median difference 1.00, IQR (0.94–1.00), pall.: p = 0.045, median difference 1.00, IQR (0.88–1.00)*), curative HRQoL information (*p = 0.045, median difference 0.29, IQR (0.00–0.45)*) and palliative side effects information (*p = 0.045, median difference 0.65, IQR (0.57–0.81)*. Also for curative cases, the relative number of natural frequencies used to indicate a treatment outcome was significantly higher after the intervention for overall outcome communication (*p < 0.001, median difference 0.11, IQR (0.04–0.15))* and survival information *(p = 0.045, median difference 0.58, IQR (0.12–0.79)*). All other secondary outcomes coded per item were not significantly different between pre- and post-intervention for either curative or palliative cases. See Fig. 4 for an overview of secondary outcomes on the item level for curative and palliative cases. HCPs assessed the training with a 7.7 and the e-learning, as part of the training, with a 7.8 averagely (1:very bad—10: very good). See Appendix 4 for more details on training evaluation.

## Discussion

In this stepped-wedge intervention study, we demonstrated that the combined utilization of a prediction tool and communication skills training significantly enhanced the precision of the overall information given about treatment outcomes in a simulated context of esophageal and gastric cancer. Specifically, for the curative simulated scenarios, HCPs’ survival information became more precise following the intervention. For the palliative simulated scenario, HCPs’ information about side effects was more precise after the intervention. Furthermore, the intervention positively influenced the personalization of outcomes to clinical characteristics and the tailoring of communication about treatment outcomes to individual patients’ needs and preferences.

Among all treatment outcomes displayed in the Source tool, the tool provides the most comprehensive and most personalized information for survival. Indeed, amongst other outcomes, the relative number of utterances supported by visualizations increased significantly in both the curative and the palliative setting for survival information. Importantly, in the curative setting, HCPs’ extensive use of the tool combined with their acquired skills and attitudes likely resulted in them informing patients about chances of survival in a significantly more precise manner than before.

For the palliative setting, inspection of the data in Fig. 3 revealed that HCPs already gave quite precise information on survival before the introduction of the intervention, leaving little room for improvement. As such, we did not observe an effect on the precision of survival information in this setting. It might be that the HCPs participating in the palliative setting, medical oncologists, were already more used to or more skilled in providing specific information about survival due to their daily clinical practice of introducing patients with treatment which has the main goal of prolonging patients’ lives. Moreover, the current simulated setting involves a highly educated patient who is not overly emotional, curious for numbers and asks for more information when the HCP talks about a difference in survival between treatment options. A consult with such a patient might lack some of the more difficult real-world challenges that clinicians face when informing patients in clinical practice. Still, the training and tool did improve HCPs’ skills and attitude in providing clinically personalized information, asking the right questions in order to tailor the information to individual needs and preferences, and supporting their information with visualizations.

The precision of side effect and complication information varied based on the setting in which it was provided by HCPs. Interestingly, in the palliative setting, there was an improvement in precision, whereas in the curative setting, no such improvement was observed. In line, a distinct pattern was found in use of visualizations, which were used significantly more at post measurements in the palliative setting, compared to pre-measurements, whereas in the curative setting no such increase was found. This divergence in tool usage might be attributed to differences in experienced clinical applicability of the data utilized for side effects in the tool. Specifically, since data from the Netherlands Cancer Registry (NCR) were not available for side effects, mainly impersonalized meta-analysis data were presented in the Source tool except for prediction models about urological complications, severe complications, and 30-day mortality. Possibly, the improvement in radiotherapy techniques since the CROSS trial and the rapid developments in surgical techniques, improving mortality and morbidity rates for treatment of potentially curable esophagogastric cancer, might lead to particularly surgical and radiation oncologists feeling the tool complication and side effect data in the curative setting are outdated.^43^ In addition, the surgical risks for anastomotic leakage, for instance, seem to vary across countries in Europe and even across centers in the Netherlands,^44^, ^45^, ^46^, ^47^, ^48^ possibly contributing to HCPs’ experience of the tool data being a poor ‘fit’ to the performance rates in their own hospital. Future engagements in enlarging the registry of morbidity data and in developing and frequently updating personalized prediction models, might enhance HCPs’ feeling of applicability of the data to specific patients and consequently encourage them to provide patients with precise information on side effects and complications.

Interestingly, in neither the curative nor the palliative setting, the precision of HRQoL information improved. However, the relative number of utterances supported by visualizations did improve significantly for curative cases. Possibly, the information that the Source tool provided for HRQoL was not sufficiently precise for HCPs to substantiate their information with numbers. HRQoL data used in the tool are those from the Dutch nationwide quality of life registry (Prospective Observational Cohort study of Oesophageal-gastric cancer Patients).^33^ Yet, the variation in the available patient samples at the time of data collection was quite high, resulting in large confidence intervals and great uncertainties about the absolute and relative differences in HRQoL between treatment options in the tool. Another explanation might be that HCPs found it more challenging to provide patients with numbers that reflect subjective experiences, as is the case with patient-reported outcome measures (PROMs), compared to more objective information, as is the case for the tool’s data on survival, side effects, and complications.^22^^,^^49^ For both of the aforementioned reasons, it might be that HCPs struggled with which message to covey to patients based on these data, as part of their usual storyline of the pros and cons of treatment options. Further research on the use of PROMs in the decision-making process should give more specific guidelines on how to use precise HRQoL information to benefit patients and their decision making.

Although the median difference in consultation duration suggested a change between pre and post intervention, we did not find a significant effect of the intervention on the overall consultation duration. This finding is in line with literature indicating that the application of shared decision making, of which precise information giving is an essential element, does not by definition require longer consultations.^50^ The finding that using the Source tool and having been trained on information giving does not necessarily prolong the duration of HCPs’ consultations with patients, may facilitate future implementation of the intervention in clinical practice. In addition, HCPs’ overall positive evaluation of the utility of the training may support its’ transfer into clinical practice.^51^ Further improvements of the tool and training will be made based on feedback provided in the current trial.

Altogether, the combined use of the Source tool and training has shown improvements in communication about treatment outcomes. While overall precision, personalization, and tailoring of outcome information improved, there is room for further advancement. Information giving regarding some outcome categories could still be more precise, and alternative ways of framing outcomes are still seldom utilized to clarify treatment information. Also, from the results of the current study, we do not know if the more precise, more personalized, and more tailored information that was provided by HCPs positively affects patients’ understanding of treatment outcomes. Therefore, patients’ comprehension of the Source tool is currently assessed in a follow-up study, particularly focusing on low health literacy patients. Nevertheless, the current results demonstrate promising effects of the tool and training on HCPs’ outcome communication skills. If proven to be effective in real-life clinical practice, the use of similar tools would be imaginable in other cancer settings. In addition, further research should explore the impact of the tool and training on how treatment options are presented and the subsequent decision-making process.

Our findings need to be interpreted in the light of some limitations. First, the simulated setting that is currently used, prevents drawing definitive conclusions for clinical practice, especially for the long term. The important next step, which is currently being undertaken as part of this stepped-wedge trial, is to test the effects of the intervention in real-life clinical consultations (NCT04232735). Another limitation is the limited statistical power that remains, when comparing the effects for the curative and palliative cases and for all outcome categories separately. Lastly, a part of the current study’s SPAs and training components have been performed in an online, video call setting due to COVID-19 regulations. For SPA measurements as well as for training, this could have influenced the perceived realism and comparability to clinical practice. Still, both of these parameters were scored as acceptable (>6.5) by HCPs themselves. However, considering the simulated nature of the study, a ‘Hawthorne effect’ of HCPs intentionally increasing their precision of information at post-measurement, could not be ruled-out.^52^ Yet, there is little evidence for an effect on behavior, when HCPs were aware of being video recorded.^53^

Some strengths of the current study also deserve mentioning. Standardization of patient characteristics and elimination of confounding by patient characteristics was allowed by using actors, who followed a script, instead of real patients. This design enabled us to establish whether HCPs are able to apply the knowledge and skills gained from the intervention, before investigating whether they can transfer these skills to the actual clinical setting. Secondly, prior to using our prediction models in the current study, these models have been proven to show acceptable to good performances, have been updated multiple times and were externally validated.^27^^,^^30^^,^^54^ In the future, these prediction models will be kept up to date by adding new data. Lastly, whereas many prediction tools have already been developed for predicting several diseases’ outcomes, not many studies have yet evaluated the effect of prediction tools on HCP’s communication. To our knowledge, this is the first study evaluating the effect of such a tool on the precision of outcome communication.

In conclusion, results of a tool and training to stimulate precise, evidence-based, personalized and tailored information giving about treatment outcomes are promising in a simulated setting. The next steps are to investigate the effect on patient-reported outcomes, such as their knowledge about the expected treatment outcomes and their evaluation of the decision, and to implement the tool and training in clinical practice.

## Contributors

L.F. van de Water: Conceptualization, data curation, formal analysis, investigation, methodology, project administration, resources, visualisation, writing—original draft.

S.C. Kuijper: Data curation, formal analysis, methodology, software, visualisation, writing–review & editing.

I. Henselmans: Conceptualization, methodology, resources, supervision, writing–review & editing.

E.N. van Alphen: Data curation, formal analysis, writing–review & editing.

E.S. Kooij: Data curation, formal analysis, investigation, writing–review & editing.

M.M. Calff: Conceptualization, resources, writing–review & editing.

L.V. Beerepoot, J. Buijsen, W.J. Eshuis, E.D. Geijsen, S.H.C. Havenith, F.F.B.M. Heesakkers, S. Mook, K. Muller, H.C. Post, H. Rütten, M. Slingerland, T. van Voorthuizen: Resources, writing–review & editing.

H.W.M. van Laarhoven: Conceptualization, funding acquisition, methodology, supervision, writing–review & editing.

E.M.A. Smets: Conceptualization, funding acquisition, methodology, supervision, writing–review & editing.

## Data sharing statement

Deidentified participant data are available on reasonable request to the authors, using a signed data access agreement. Please contact H.W.M. van Laarhoven, h.vanlaarhoven@amsterdamumc.nl. Study protocol is available at NCT04232735.

## Declaration of interests

M. Slingerland: Advisory role: Lilly, AstraZeneca en BMS.

H.W.M. van Laarhoven: Financial support by the Dutch Cancer Society, Bayer, BMS, Celgene, Janssen, Incyte, Eli Lilly, MSD, Nordic Pharma, Philips, Roche, Servier. Consulting fees from Amphera, AstraZeneca, Beigene, BMS, Daiichy-Sankyo, Dragonfly, Eli Lilly, MSD, Nordic Pharma, Servier. Spealer role: Astellas, Benecke, Daiichy-Sankyo, JAAP, Medtalks, Novartis, Travel Congress Management B.V.

The other authors declare no conflicts of interest.

## Appendix ASupplementary data

Supplementary Appendix

Protocol Source Trial
