# Supplementary material for: Effect of a prediction tool and communication skills training on communication of treatment outcomes: a multicenter stepped wedge clinical trial (the SOURCE trial)
Source: eClinicalMedicine. 2023 Sep 25;64:102244. doi: 10.1016/j.eclinm.2023.102244 (PMC10539636; doi:10.1016/j.eclinm.2023.102244)
Supplement: Supplementary Appendix [file mmc1.pdf]

Supplementary material – Appendices

Table of contents

Page nr.	Appendix nr.	Heading
2-3	APPENIDIX 1	Coding with the Outcome Information Scale - Oesophageal and gastric cancer (OIS-OG)
4	APPENIDIX 2	Standardized Patient Assessment (SPA) cases
5-23	APPENIDIX 3	Overview of the type of items coded to be discussed by HCPs during SPAs
24-25	APPENIDIX 4	HCP evaluation of the e-learning and training

APPENDIX 1: Coding with the Outcome Information Scale - Oesophageal and gastric cancer (OIS-OG)

Instrument

The OIS-OG was developed using treatment information consultations of the SOURCE pilot study and from trials on shared decision making (SDM) and informing patients with a similar design and patient population (19, 27, 48). Using input from these consultations, iterative development (using multiple development and test phases) took place. After each subset of consultations (2-5), coders and experts compared their scores and discussed inconsistencies to arrive at a common interpretation of the variables and their levels. If required, the manuals were complemented with directions to improve clarity and consensus. Development of the scale was frequently evaluated by the principal investigators. The development process resulted in a coding manual with detailed descriptions of the primary and secondary outcomes including several examples.

For the primary outcome, precision of the outcome information, development resulted in a 4-level Likert scale for the outcome categories survival, side effects and complications and response and recurrence: 1. Non-informative (e.g. "I don't know"), 2. Absolute statement with little or no nuance or detail about risks (e.g. "Treatment A has better survival chances than treatment B", "One of the side effects is nausea") 3. Nuanced statements with some detail about risks, but no actual numbers (e.g. "It's months rather than years", "A lot of people get nauseous") 4. Statements with numbers (e.g. "50% of people"). The scale for health-related quality of life (HRQoL) was comprised of 3 levels: 1. Non-informative (e.g. "I don't know"), 2. Absolute statement with little or no nuance or detail about risks (e.g. "With treatment A you'd have better HRQoL than with treatment B) 3. Nuanced statements with some detail about risks, and statements with actual numbers (e.g. "right after surgery patient reported HRQoL drops, but after a few weeks it improves again").

Each utterance regarding treatment outcome information was coded in a coding framework, comprised of a list of all possible treatment outcomes divided per treatment option and/or symptom. Thus, separate coding frameworks were developed for curative and palliative scenarios. The coding frameworks for 'survival' and 'response and recurrence' were based on a list of all treatment options coded in the development consultations. For 'side effects and complications' and 'HRQoL' the coding frameworks were based on the CTCAE guidelines, functional disorder taxonomy and EORTC quality of life scales, and was complemented with input from the development consultations (5, 49-51). See Appendix Table 1 for an example of one of the coding frameworks. However, as the frameworks for 'side effects and complications' and 'HRQoL' showed several overlapping topics, it was decided to, in the case of an overlapping topic, define side effects as short-term outcomes and strictly caused by the treatment and quality of life as long term outcomes and/or caused by the disease itself.

Secondary outcomes initiative, timeframe, visualization, percentage, natural frequency, framing, uncertainty communication and clinical trial treatment were coded for each utterance of outcome information, see Appendix Table 1. Personalizing to clinical characteristics, checking of current knowledge/understanding and tailoring to information needs/preferences were coded on a consultation level, by counting the number of remarks.

Coding process

Development consultations were used for calibration between the 3 coders, 2 MSc. in Psychology (LvdW and EK) and 1 MSc. in Anthropology (EvA). When no significant changes to the coding manual were made, coders proceeded to analyzing the trial consultations.

Trial consultations were coded independently in sets of 20 consultations. By randomization and renaming of the consultations, coders were blinded for the condition of the consultation (pre- or post-intervention). Each consultation was coded by two coders. After each set, coders discussed inconsistencies and doubts until consensus was reached and complemented the coding manual when necessary and previously coded consultations were reassessed. After initial coding of secondary outcomes 'personalization', 'current knowledge checks' an 'tailoring attempt', LvdW re-read and compared coded fragments to reach the fragments coded for these three variables. All secondary variables coded at the utterance level were analyzed in the same manner as the primary outcome, using frequencies and rescaling them to a scale from 0-100.

Appendix1. Table 1. Cut-out of the coding framework used by the Outcome Information Scale, for three side effects of chemotherapy

	Maximal precision score (primary)	*Initiative (HCP/SP)*	*Timeframe (0/1)*	*Visualization (0/1)*	*Percentage (0/1)*	*Natural frequency (0/1)*	*Framing (0/1)*	*Uncertainty communication (0/1)*	*Clinical trial treatment (0/1)*
**Side effects of chemotherapy**									
**Nausea**	2	HCP	1	0	0	0	0	0	0
**Fatigue**	-	-	-	-	-	-	-	-	-
**Diarrhea/ changed stool**	3	Patient	0	0	0	0	1	1	0

Statistical analysis

Scores on the primary outcome were transformed previous to statistical analyses, see Method section. To transform a scale score *x* from a range of [min(x),max(x)] to a range of [a,b], we used the standard transformation formula:

$$x_{normalized} = (b-a)\frac{x - min(x)}{max(x) - min(x)} + a$$

Min(x) and max(x) were determined as the theoretical minimum and maximum scale score on which a particular HCP scored.

For example, if three Likert items were scored, each with a scale of [1,4], the theoretical minimum and maximum range of the three items combined was [4,12]. The value of *x* on this range was transformed to a [1,100] scale.

APPENDIX 2: Standardized Patient Assessment (SPA) cases

	Palliative case 1	Palliative case 2	Curative case 1	Curative case 2
**Sex**	Male	Male	Male	Male
**Age**	58	63	76	74
**Occupation**	Accountant	Retired soil engineer	Retired accountant	Retired soil engineer
**Type of cancer**	Metastasized gastric	Metastasized oesophageal	Localized oesophageal	Localized oesophageal
**Indicated treatment**	Palliative systemic treatment	Palliative systemic treatment	Curative treatment; fit for surgery	Curative treatment; fit for surgery
**Tumor**	Adenocarcinoma with shoulder bone metastases	Adenocarcinoma with lung metastases	Squamous cell carcinoma	Squamous cell carcinoma
**TNM**	T2N2M1	T1N0M1	T3N1M0	T3N1M0
**WHO**	WHO-1	WHO-1	WHO-1	WHO-1
**Comorbidities**	Hypertension	Hypertension	Hypertension; heart attack (10 years ago)	Hypertension; type 2 diabetes; arthrosis

APPENDIX 3: Overview of the type of items coded to be discussed by HCPs during SPAs

Appendix 3. Table 1. Items coded at post-intervention palliative SPAs for medical oncologists(O1-O12). X=coded by one or both of the coders

	O1	O2	O3	O4	O5	O6	O7	O8	O9	O10	O11	O12
**Survival**												
Survival pall. chemotherapy	x	x	x	x	x	x	x	x	x	x	x	x
Survival Best Supportive Care	x	x	x		x		x	x	x		x	x
Survival pall. radiotherapy												
Survival immunotherapy												
**Side effects**												
Side effects pall. chemoth. - in general	x	x	x	x	x	x	x	x	x	x		x
Alopecia/hair loss				x	x	x					x	
Fatigue		x	x	x	x		x	x	x	x	x	
Nausea/vomiting	x		x	x	x	x	x	x		x	x	x
Decreased appetite		x				x				x		
Weight loss	x											
Thrombocytopaenia				x			x			x		
Anaemia	x	x	x							x		
Leukopaenia/ (febrile) neutropaenia	x		x	x	x	x	x	x		x	x	x
Allergic reaction	x	x								x	x	
Changes in stool	x		x		x	x		x	x	x	x	x
Skin complaints	x			x	x	x		x	x		x	x
Tingling/neuropathy	x	x	x	x	x	x	x	x	x	x	x	x
Mucosal inflammation										x		
Stomatitis										x		
Taste changes			x		x							
Hospitalization	x						x			x		
Side effects immunoth. - in general												
Skin complaints												
Fatigue												
Changes in stool												
Decreased disease resistance												
Side effects pall. radiotherapy - in general												
Side effects Best Supp. Care - in general						x						
**Health-Related Quality of Life (HRQoL)**												
Overall HRQoL pall. chemoterapy	x	x	x	x	x	x	x	x	x	x	x	x
Overall HRQoL Best Supp. Care	x				x	x	x		x	x		
Overall HRQoL pall. radioterapy									x			
Overall HRQoL immunoterapy												
Physical functioning	x	x		x	x		x			x		
Social functioning		x										
Role functioning				x								
Cognitive functioning												
Emotional functioning						x			x			
Diarrhoea												
Hair loss												
Nausea/vomiting							x					x

Appetite loss												
Fatigue			x						x	x		
Reflux												
Dry mouth												
Weight loss	x			x								
Trouble with coughing										x		
Pain/discomfort	x		x	x	x	x	x		x	x		x
Odynophagia												
Dysphagia	x								x	x		
Trouble with taste			x		x	x					x	x
Trouble swallowing saliva												
Choking when swallowing												
Constipation												
Shortness of breath	x											
Eating restriction			x	x	x	x				x		x
Trouble talking												
Insomnia												
Anxiety												
**Response and recurrence**												
Response/recurrence pall. chemoth.	x	x	x	x	x	x	x	x	x	x	x	x
Response/recurrence Best Supp. Care						x				x		
Response/recurrence pall. radiotherapy										x		
Response/recurrence immunotherapy												

Appendix 3. Table 2. Items coded at post-intervention palliative SPAs for medical oncologists (O1-O12). X=coded by one or both of the coders

	O1	O2	O3	O4	O5	O6	O7	O8	O9	O10	O11	O12
**Survival**												
Survival pall. chemotherapy	x	x	x	x	x	x	x	x	x	x	x	x
Survival Best Supportive Care	x	x	x	x	x	x	x	x	x	x	x	x
Survival pall. radiotherapy												
Survival immunotherapy												
**Side effects**												
Side effects pall. chemoth. - in general	x	x	x	x	x	x	x	x	x	x	x	x
Alopecia/hair loss										x		
Fatigue	x	x	x	x				x		x	x	
Nausea/vomiting	x	x	x	x	x	x	x	x		x	x	x
Decreased appetite	x	x	x		x	x	x	x		x	x	x
Weight loss						x						
Thrombocytopaenia												
Anaemia												
Leukopaenia/ (febrile) neutropaenia	x		x	x			x	x			x	x
Allergic reaction										x		
Changes in stool	x	x	x					x	x			
Skin complaints	x	x	x		x	x	x	x	x	x	x	x
Tingling/neuropathy	x	x	x	x	x	x		x	x	x	x	x
Mucosal inflammation	x							x				
Stomatitis	x							x				
Taste changes		x	x				x			x	x	x
Hospitalization		x	x						x	x		
Side effects immunoth. - in general												
Skin complaints												
Fatigue												
Changes in stool												
Decreased disease resistance												
Side effects pall. radiotherapy - in general												
Side effects Best Supp. Care - in general												
**Health-Related Quality of Life (HRQoL)**												
Overall HRQoL pall. chemoterapy	x	x	x	x	x	x	x		x	x	x	x
Overall HRQoL Best Supp. Care	x	x		x	x		x		x			
Overall HRQoL pall. radioterapy							x					x
Overall HRQoL immunoterapy												
Physical functioning		x		x	x	x	x		x			x
Social functioning				x	x	x						x
Role functioning	x	x		x	x	x	x					x
Cognitive functioning												
Emotional functioning					x							
Diarrhoea												
Hair loss												
Nausea/vomiting												x
Appetite loss		x			x						x	
Fatigue					x					x	x	

Reflux												
Dry mouth												
Weight loss												
Trouble with coughing					x							
Pain/discomfort	x	x		x	x		x			x	x	x
Odynophagia												
Dysphagia	x			x						x		
Trouble with taste		x	x		x						x	x
Trouble swallowing saliva												
Choking when swallowing												
Constipation					x							
Shortness of breath					x							
Eating restriction		x	x		x		x			x		
Trouble talking												
Insomnia												
Anxiety					x							
**Response and recurrence**												
Response/recurrence pall. chemoth.	x	x	x	x	x	x	x	x	x	x	x	x
Response/recurrence Best Supp. Care	x								x	x		x
Response/recurrence pall. radiotherapy							x					
Response/recurrence immunotherapy												

Appendix 3. Table 3. Items coded at pre-intervention curative SPAs for radiation oncologists (R1-R8). X=coded by one or both of the coders

	R1	R2	R3	R4	R5	R6	R7	R8
**Survival**								
Survival Neo-adj. chemoradiatiotherapy+ surgery	x	x	x	x	x	x	x	x
Survival Definitive chemoradiotherapy	x	x	x		x		x	
Survival Best Supportive Care		x	x			x		
Survival Surgery only		x				x		x
Survival Neo-adj. chemoradiatiotherapy + wait-and-see								
Survival Perioperative chemoradiatiotherapy								
**Side effects and complications**								
Side effects/compl. - neo-adj. chemoradiotherapy + surgery – in general		x	x	x				
Side effects/compl. - Definitive chemoradiotherapy – in general	x	x	x		x		x	
Side effects/compl. – Best Supportive Care – in general		x						
Side effects/compl. – Surgery only – in general								
Side effects/compl. - neo-adj. chemoradiotherapy + wait-and-see – in general								
Side effects/compl. - Perioperative chemoradiotherapy – in general								
Complications surgery – in general		x	x	x	x	x	x	
Anastomotic leakage								
Pneumonia/pulmonary complaints								
Tachyarrhythmia								
Lymfocele								
Infection/mediastinitis								
Wound infection								
Wongenezing stoornis								
Abces								
Urologische complicaties								
Bleeding								
Vocal difficulties								
Weight loss								
Thrombosis								
Additional surgery								
Death from surgery (30 days)								
Death from surgery (90 days)								
Hopitalization								
Hopitalization ICU								
Intestinal blokcade								

Gastro-intestinal complications								
Severe complications								
Blood count/increased chance of infections								
Pain								
Side effects chemotherapy – in general		x	x	x		x		x
Alopecia/hair loss				x	x			
Fatigue	x			x	x	x		
Nausea/vomiting		x		x			x	
Appetite loss				x				
Weight loss								
Thrombocytopaenia		x						
Anaemia		x						
Leukopaenia/ (febrile) neutropaenia		x						
Changes in stool								
Skin complaints								
Tingling/neuropathy								
Mucosal inflammation								
Stomatitis								
Taste changes	x			x			x	
Hospitalization								
Changes in blood pressure		x						
Side effects radiotherapy – in general	x	x	x	x	x	x	x	x
Weight loss	x	x	x	x		x	x	
Dysphagia/pain/irritation	x	x	x	x	x	x	x	x
Fever								
Fatigue	x	x	x	x	x	x	x	x
Nausea/vomiting		x	x	x	x		x	
Appetite loss				x				
Hospitalization								
Skin complaints				x	x			
Pain in the lower part of the esophagus						x		x
Pulmonary complaints								
Stenosis		x			x	x		
Changes in blood pressure		x						
**Health-Related Quality of Life (HRQoL)**								
Overall HRQoL Neoadj. chemoradiotherapy + surgery				x			x	
Overall HRQoL Definitive chemoradiatherapy								

Overall HRQoL Best Supportive Care								
Overall HRQoL Surgery only								
Overall HRQoL Neoadj. chemoradiotherapy + wait-and-see								
Overall HRQoL Perioperative chemoradiotherapy								
Reflux/sleeping upright				x				
Stomach reduction	x		x	x			x	
Dumping								
Changes in taste								
Stenosis					x			
Trouble with gall								
Diarrhoea (from dumping)								
Physical functioning	x			x	x			
Social functioning				x				
Role functioning								
Cognitive functioning	x		x					
Emotional functioning								
Diarrhoea								
Hair loss								
Nausea/vomiting								
Appetite loss								
Fatigue								
Reflux				x				
Dry mouth								
Weight loss								
Trouble with coughing								
Pain/discomfort				x				
Odynophagia								
Dysphagia		x	x					
Trouble with taste	x			x			x	
Trouble swallowing saliva								
Choking when swallowing								
Constipation								
Shortness of breath								
Eating restriction			x	x	x	x	x	
Trouble talking								
Insomnia								
Anxiety								

**Response and recurrence**								
Response/recurrence Neo-adj. chemoradiatiotherapy+ surgery	x	x		x	x	x	x	x
Response/recurrence Definitive chemoradiotherapy		x	x		x		x	
Response/recurrence Best Supportive Care								
Response/recurrence Surgery only					x	x		
Response/recurrence Neo-adj. chemoradiatiotherapy + wait-and-see					x	x		
Response/recurrence Perioperative chemoradiatiotherapy								

Appendix 3. table 4. Items coded at post-intervention curative SPAs for radiation oncologists (R1-R8). X=coded by one or both of the coders

	R1	R2	R3	R4	R5	R6	R7	R8
**Survival**								
Survival Neo-adj. chemoradiatiotherapy+ surgery	x	x	x	x	x	x	x	x
Survival Definitive chemoradiotherapy	x	x	x	x	x	x	x	x
Survival Best Supportive Care								
Survival Surgery only								
Survival Neo-adj. chemoradiatiotherapy + wait-and-see								
Survival Perioperative chemoradiatiotherapy								
**Side effects and complications**								
Side effects/compl. - neo-adj. chemoradiotherapy + surgery – in general	x	x	x	x	x	x	x	
Side effects/compl. - Definitive chemoradiotherapy – in general		x	x	x	x		x	
Side effects/compl. – Best Supportive Care – in general								
Side effects/compl. – Surgery only – in general								
Side effects/compl. - neo-adj. chemoradiotherapy + wait-and-see – in general								
Side effects/compl. - Perioperative chemoradiotherapy – in general								
Complications surgery – in general			x	x	x		x	x
Anastomotic leakage			x			x	x	x
Pneumonia/pulmonary complaints			x			x	x	x
Tachyarrhythmia								
Lymfocele								
Infection/mediastinitis							x	
Wound infection			x				x	
Wongenezing stoornis								
Abces								
Urologische complicaties								
Bleeding								
Vocal difficulties								
Weight loss			x					
Thrombosis								
Additional surgery						x	x	x
Death from surgery (30 days)						x	x	x
Death from surgery (90 days)								
Hopitalization							x	x

Hopitalization ICU								
Intestinal blokcade								
Gastro-intestinal complications								
Severe complications								
Blood count/increased chance of infections								
Pain								
Side effects chemotherapy – in general		x		x				x
Alopecia/hair loss								
Fatigue	x			x			x	x
Nausea/vomiting	x	x					x	x
Appetite loss	x						x	x
Weight loss			x				x	
Thrombocytopaenia		x						
Anaemia								
Leukopaenia/ (febrile) neutropaenia		x						
Changes in stool								
Skin complaints								
Tingling/neuropathy								
Mucosal inflammation								
Stomatitis								
Taste changes	x	x						
Hospitalization								
Changes in blood pressure						x		
Side effects radiotherapy – in general	x		x	x	x		x	x
Weight loss			x	x	x		x	
Dysphagia/pain/irritation	x	x	x	x	x	x		x
Fever								
Fatigue	x	x	x	x	x	x	x	x
Nausea/vomiting	x	x	x				x	x
Appetite loss	x						x	x
Hospitalization								
Skin complaints								
Pain in the lower part of the esophagus								
Pulmonary complaints								
Stenosis		x			x	x		

Changes in blood pressure						x		
**Health-Related Quality of Life (HRQoL)**								
Overall HRQoL Neoadj. chemoradiotherapy + surgery		x	x	x		x	x	x
Overall HRQoL Definitive chemoradiatherapy		x		x		x	x	
Overall HRQoL Best Supportive Care								
Overall HRQoL Surgery only								
Overall HRQoL Neoadj. chemoradiotherapy + wait-and-see								
Overall HRQoL Perioperative chemoradiotherapy								
Reflux/sleeping upright					x			x
Stomach reduction			x		x	x	x	x
Dumping								
Changes in taste								
Stenosis								
Trouble with gall								
Diarrhoea (from dumping)								
Physical functioning			x	x	x		x	x
Social functioning				x			x	x
Role functioning						x	x	
Cognitive functioning								
Emotional functioning			x					
Diarrhoea								
Hair loss								
Nausea/vomiting								
Appetite loss								x
Fatigue								
Reflux								
Dry mouth								
Weight loss								
Trouble with coughing								
Pain/discomfort								x
Odynophagia								
Dysphagia			x					
Trouble with taste		x				x		x
Trouble swallowing saliva								
Choking when swallowing								

Constipation								
Shortness of breath								
Eating restriction			x	x		x		
Trouble talking								
Insomnia								
Anxiety			x			x		
**Response and recurrence**								
Response/recurrence Neo-adj. chemoradiatiotherapy+ surgery	x	x	x	x	x	x	x	x
Response/recurrence Definitive chemoradiotherapy		x	x	x	x			
Response/recurrence Best Supportive Care								
Response/recurrence Surgery only								
Response/recurrence Neo-adj. chemoradiatiotherapy + wait-and-see								
Response/recurrence Perioperative chemoradiatiotherapy								

Appendix 3. table 5. Items coded at pre-intervention curative SPAs for surgical oncologists (S1-S11). X=coded by one or both of the coders

	S1	S2	S3	S4	S5	S6	S7	S8	S9	S10	S11
**Survival**											
Survival Neo-adj. chemoradiatiotherapy+ surgery	x	x	x	x	x	x	x	x	x	x	x
Survival Definitive chemoradiotherapy	x	x		x			x	x	x	x	x
Survival Best Supportive Care			x		x		x	x	x	x	
Survival Surgery only											
Survival Neo-adj. chemoradiatiotherapy + wait-and-see			x				x	x			
Survival Perioperative chemoradiatiotherapy											
**Side effects and complications**											
Side effects/compl. - neo-adj. chemoradiotherapy + surgery – in general				x		x	x	x			x
Side effects/compl. - Definitive chemoradiotherapy – in general										x	
Side effects/compl. – Best Supportive Care – in general							x				
Side effects/compl. – Surgery only – in general											
Side effects/compl. - neo-adj. chemoradiotherapy + wait-and-see – in general											
Side effects/compl. - Perioperative chemoradiotherapy – in general											
Complications surgery – in general	x	x	x	x	x		x	x	x	x	x
Anastomotic leakage	x	x	x	x				x	x	x	x
Pneumonia/pulmonary complaints	x	x	x	x				x	x	x	x
Tachyarrhythmia	x		x					x		x	x
Lymfocele	x									x	
Infection/mediastinitis	x	x									
Wound infection								x		x	
Wongenezing stoornis											
Abces								x			
Urologische complicaties										x	
Bleeding	x							x			
Vocal difficulties			x								
Weight loss	x									x	
Thrombosis			x								
Additional surgery	x	x		x					x	x	
Death from surgery (30 days)	x			x			x		x	x	
Death from surgery (90 days)											
Hopitalization			x	x				x	x		

Hopitalization ICU	x	x		x					x	x	
Intestinal blokcade											
Gastro-intestinal complications											
Severe complications	x										
Blood count/increased chance of infections	x										
Pain			x				x			x	
Side effects chemotherapy – in general	x				x	x		x			
Alopecia/hair loss								x			
Fatigue							x	x		x	
Nausea/vomiting											
Appetite loss											
Weight loss						x		x			
Thrombocytopaenia											
Anaemia											
Leukopaenia/ (febrile) neutropaenia								x			
Changes in stool								x			
Skin complaints								x			
Tingling/neuropathy							x				
Mucosal inflammation										x	
Stomatitis											
Taste changes							x	x			
Hospitalization								x			
Changes in blood pressure											
Side effects radiotherapy – in general	x							x			
Weight loss					x	x					
Dysphagia/pain/irritation					x	x	x	x		x	
Fever								x			
Fatigue							x			x	
Nausea/vomiting											
Appetite loss											
Hospitalization								x			
Skin complaints											
Pain in the lower part of the esophagus							x				
Pulmonary complaints											
Stenosis										x	

Changes in blood pressure											
**Health-Related Quality of Life (HRQoL)**											
Overall HRQoL Neoadj. chemoradiotherapy + surgery	x	x	x	x	x	x	x	x	x	x	x
Overall HRQoL Definitive chemoradiatherapy		x	x	x				x		x	
Overall HRQoL Best Supportive Care		x					x				
Overall HRQoL Surgery only											
Overall HRQoL Neoadj. chemoradiotherapy + wait-and-see					x		x				
Overall HRQoL Perioperative chemoradiotherapy											
Reflux/sleeping upright	x		x	x	x			x		x	x
Stomach reduction	x	x	x	x	x		x	x	x	x	x
Dumping			x								
Changes in taste							x				
Stenosis	x	x									
Trouble with gall											
Diarrhoea (from dumping)			x								
Physical functioning	x	x	x	x	x	x	x		x		x
Social functioning				x							
Role functioning											
Cognitive functioning											
Emotional functioning						x					
Diarrhoea											
Hair loss											
Nausea/vomiting											
Appetite loss							x				
Fatigue											
Reflux			x								
Dry mouth											
Weight loss										x	
Trouble with coughing											
Pain/discomfort											x
Odynophagia											
Dysphagia		x					x			x	
Trouble with taste											
Trouble swallowing saliva											
Choking when swallowing											

Constipation											
Shortness of breath											
Eating restriction	x		x	x			x	x		x	x
Trouble talking											
Insomnia											
Anxiety											
**Response and recurrence**											
Response/recurrence Neo-adj. chemoradiatiotherapy+ surgery	x	x	x	x	x	x	x	x	x	x	
Response/recurrence Definitive chemoradiotherapy			x	x			x	x	x	x	
Response/recurrence Best Supportive Care			x				x		x		
Response/recurrence Surgery only						x		x			
Response/recurrence Neo-adj. chemoradiatiotherapy + wait-and-see			x		x	x	x	x			
Response/recurrence Perioperative chemoradiatiotherapy											

Appendix 3. table 6. Items coded at post-intervention curative SPAs for surgical oncologists (S1-S11). X=coded by one or both of the coders

	S1	S2	S3	S4	S5	S6	S7	S8	S9	S10	S11
**Survival**											
Survival Neo-adj. chemoradiatiotherapy+ surgery	x	x	x	x	x	x	x	x	x	x	x
Survival Definitive chemoradiotherapy	x	x	x	x	x	x	x	x	x	x	x
Survival Best Supportive Care	x	x		x	x	x	x	x	x		
Survival Surgery only							x	x	x		
Survival Neo-adj. chemoradiatiotherapy + wait-and-see											
Survival Perioperative chemoradiatiotherapy											
**Side effects and complications**											
Side effects/compl. - neo-adj. chemoradiotherapy + surgery – in general				x			x	x			x
Side effects/compl. - Definitive chemoradiotherapy – in general		x		x						x	x
Side effects/compl. – Best Supportive Care – in general											
Side effects/compl. – Surgery only – in general											
Side effects/compl. - neo-adj. chemoradiotherapy + wait-and-see – in general										x	
Side effects/compl. - Perioperative chemoradiotherapy – in general											
Complications surgery – in general	x	x	x	x	x	x	x	x		x	

Anastomotic leakage	x	x	x	x	x	x	x	x		x	x
Pneumonia/pulmonary complaints	x	x	x	x	x	x	x	x		x	x
Tachyarrhythmia										x	x
Lymfocele						x				x	
Infection/mediastinitis											
Wound infection						x				x	
Wongenezing stoornis											
Abces											
Urologische complicaties										x	
Bleeding											
Vocal difficulties											
Weight loss								x			
Thrombosis						x					
Additional surgery	x	x	x							x	
Death from surgery (30 days)	x	x		x		x	x			x	
Death from surgery (90 days)											
Hopitalization			x	x	x		x	x		x	
Hopitalization ICU	x	x				x				x	x
Intestinal blokcade											
Gastro-intestinal complications							x	x			
Severe complications		x			x						
Blood count/increased chance of infections											
Pain						x			x		
Side effects chemotherapy – in general											x
Alopecia/hair loss								x			
Fatigue	x	x		x				x			
Nausea/vomiting	x			x				x			
Appetite loss	x			x							
Weight loss	x							x			
Thrombocytopaenia											
Anaemia											
Leukopaenia/ (febrile) neutropaenia											
Changes in stool								x			
Skin complaints								x			
Tingling/neuropathy											
Mucosal inflammation											
Stomatitis											

Taste changes								x			
Hospitalization	x										
Changes in blood pressure											
Side effects radiotherapy – in general			x								
Weight loss	x							x			
Dysphagia/pain/irritation					x			x			
Fever											
Fatigue	x	x		x	x	x					
Nausea/vomiting	x			x							
Appetite loss	x			x							
Hospitalization	x							x			
Skin complaints											
Pain in the lower part of the esophagus											
Pulmonary complaints							x				
Stenosis										x	
Changes in blood pressure											
**Health-Related Quality of Life (HRQoL)**											
Overall HRQoL Neoadj. chemoradiotherapy + surgery	x	x	x	x	x		x	x	x	x	x
Overall HRQoL Definitive chemoradiatherapy	x				x				x	x	
Overall HRQoL Best Supportive Care				x	x		x		x		
Overall HRQoL Surgery only							x				
Overall HRQoL Neoadj. chemoradiotherapy + wait-and-see											
Overall HRQoL Perioperative chemoradiotherapy											
Reflux/sleeping upright	x	x	x				x			x	x
Stomach reduction	x	x	x	x	x		x	x	x	x	x
Dumping			x		x						
Changes in taste										x	
Stenosis											
Trouble with gall							x				
Diarrhoea (from dumping)			x		x						
Physical functioning	x	x		x	x			x			
Social functioning		x						x		x	
Role functioning	x	x						x			
Cognitive functioning											
Emotional functioning		x						x			
Diarrhoea											

Hair loss											
Nausea/vomiting		x									
Appetite loss								x	x	x	
Fatigue								x			
Reflux											
Dry mouth											
Weight loss				x						x	
Trouble with coughing		x						x			
Pain/discomfort			x			x					
Odynophagia											
Dysphagia		x						x			
Trouble with taste									x	x	
Trouble swallowing saliva								x			
Choking when swallowing											
Constipation											
Shortness of breath						x					
Eating restriction	x	x	x	x	x	x	x	x	x	x	x
Trouble talking											
Insomnia											
Anxiety								x			
**Response and recurrence**											
Response/recurrence Neo-adj. chemoradiatiotherapy+ surgery	x	x	x	x	x	x	x	x	x	x	x
Response/recurrence Definitive chemoradiotherapy					x		x	x	x	x	x
Response/recurrence Best Supportive Care					x		x				
Response/recurrence Surgery only								x	x		
Response/recurrence Neo-adj. chemoradiatiotherapy + wait-and-see					x			x	x		
Response/recurrence Perioperative chemoradiatiotherapy											

APPENDIX 4: HCP evaluation of the e-learning and training

Appendix 4. Table 1. HCPs' rating of the training (T2)[1]

Construct (N=31)	Mean rating (M)
**Face-to-face group sessions**	
Overall rating (1-10)	7·903
Useful (1-10)	7·742
Helpful for clinical practice (1-10)	7·484
**Teacher**	
Overall rating (1-10)	8·323
**Covered theory**	
Useful (1-10)	7·581
**Practice with simulated patients**	
Instructive (1-10)	7·677
Realistic (1-10)	7·258
**Individual booster feedback session**	
Useful (1-10)	7·355
Helpful for clinical practice (1-10)	7·258
**Entire training**	
Overall grade (1-10)	7·726

[1]The evaluation questionnaire was complemented with extra qualitative items on time efficiency and separate training elements. These data were used for further improvement of the training.

Appendix 4. Table 2. HCPs' evaluation of the e-learning as part of the training (T1)[2]

Construct (N=29)[1]	Mean rating (M)
**In my opinion, the e-learning was:**	
Instructive (1-7)	5·241
Time efficient (1-7)	5·241
Boring (1-7)	3·103
Usable (1-7)	5·621
Childish (1-7)	2·793
Aesthetically pleasing (1-7)	5·241
Clear (1-7)	5·897
**It enlarged my knowledge about:**	
Informing patients in the context of a treatment decision (1-7)	5·241
The 'Source' tool (1-7)	5·690
Communicating risks and benefits (1-7)	5·103
**It was helpful for clinical practice (1-7)**	5·520
**Overall grade (1-10)**	7·821
Construct (N=29)[1]	Frequency
**Completed the e-learning**	29
**I think colleagues would use it**	
Yes	17
No	1
Maybe	11

[1]1 HCP did not complete the e-learning, 1 HCP questionnaire was missing

[2]The evaluation questionnaire was complemented with extra qualitative and quantitative items on usability, technical issues and usefulness of separate elements. These data were used for further improvement of the e-learning.
