# Supplementary material for: Effect of a prediction tool and communication skills training on communication of treatment outcomes: a multicenter stepped wedge clinical trial (the SOURCE trial)
Source: eClinicalMedicine. 2023 Sep 25;64:102244. doi: 10.1016/j.eclinm.2023.102244 (PMC10539636; doi:10.1016/j.eclinm.2023.102244)
Supplement: Protocol Source Trial [file mmc2.pdf]

SOurCE

***S**timulating evidence based, personalized and tailored information provision to improve decision making after **O**esophagogastric **C**anc**E**r diagnosis*

- a pragmatic Stepped Wedge Trial-

Version 2.5

January, 27, 2021

Short title	SOURCE
Version	2.5 January 2021
Project leader	Prof. Dr. H.W.M. Van Laarhoven<br>Dept. of Medical Oncology<br>Amsterdam UMC, location AMC<br>Meibergdreef 9; F4-224<br>1105 AZ Amsterdam<br>tel: +31 (0)20-5665955<br>h.vanlaarhoven@amsterdamumc.nl
Coordinating investigators	Prof. Dr. E.M.A. Smets<br>Dept. of Medical Psychology<br>Amsterdam UMC, location AMC<br>Meibergdreef 9; J3-220<br>1105 AZ Amsterdam<br>e.m.smets@amsterdamumc.nl<br><br>Prof. Dr. M.A.G. Sprangers<br>Dept. of Medical Psychology<br>Amsterdam UMC, location AMC<br>Meibergdreef 9; J3-221<br>1105 AZ Amsterdam<br>m.a.sprangers@amsterdamumc.nl<br><br>Drs. L.F. van de Water<br>Dept. of Medical Psychology<br>Amsterdam UMC, location AMC<br>Meibergdreef 9; J3-219<br>1105 AZ Amsterdam<br>l.f.vandewater@amsterdamumc.nl
Sponsor (in Dutch: verrichter/opdrachtgever)	AMC (Amsterdam UMC)

1. SUMMARY

The overarching aim of the programme 'Stimulating evidence based, personalized and tailored information provision to improve decision making after oesophagogastric cancer diagnosis' (SOURCE) is to provide oesophagogastric cancer patients at all disease stages with evidence based and personalized information about survival, treatment-related side-effects and/or complications and health related quality of life, tailored to patients' specific information needs, to facilitate informed decision making about treatment and thereby optimize personal care and outcomes.
For this purpose the Source tool and training were designed. The Source tool is a prediction model based website to be used by care givers for informing patients about the outcomes of treatment. The Source training for care givers is designed to learn care givers how to inform patients effectively, especially about the outcomes of treatment.

The primary aim of the SOURCE trial is to investigate the effect of the tool and training on the (numerical) precision of information about outcomes of treatment in the treatment information consultation. Secondary outcomes include: patients' satisfaction, evaluation and knowledge of the information provided by the health care provider, evaluation of the decision made and health related quality of life.

A pragmatic stepped wedge design will be used to test the effect of intervention. 21 health care providers will include 3 patients as control measurements (before intervention) and 3 patients as intervention measurements (after intervention). Participating centers will be divided into geographical subgroups, in which the transition period (in which the intervention will take place) will be spread across time.

All oesophageal and gastric cancer patients scheduled for a treatment information consultation with a participating health care provider are suitable for inclusion and will be approached for participation in the SOURCE study. Measurements include audio recordings of the treatment information consultation and questionnaires filled in by patients and health care providers at different moments in time. Audio recordings will be scored and analyzed based on a study specific coding scheme.
We estimate the physical burden/risk of this study to be negligible.

2. INTRODUCTION AND RATIONALE

Oesophagogastric cancer is a deadly disease with an increasing incidence (www.cijfersoverkanker.nl). Despite some improvement in survival, curative surgery results in a long-lasting deterioration in health related quality of life (HRQL).[1] In the palliative setting, half of the patients experience a loss in HRQL in the first 6 months after the start of chemotherapy.[2] Hence, improved survival may come with deterioration of HRQL. In the curative setting, patients consider quality of life of primary importance and they report it may outweigh survival when deciding about treatment options.[3] Likewise, one in four patients with advanced cancer indicates that sustained or improved HRQL is their main treatment goal.[4] To make well-informed treatment choices that match with patients' preferences, information about treatment outcomes, in terms of survival, treatment-related morbidity and HRQL is required. Currently, however, we lack good quality outcome data on

oesophagogastric cancer that could be used to provide patients with personalized information, accommodating the specifics of their clinical and sociodemographic situation.[5] Also, physician's skills in providing evidence based information tailored to the specific information needs of patients merit improvement.[6,7] Thus, we are unable to effectively provide oesophagogastric cancer patients with personalized information on treatment outcomes to facilitate shared decision making.

The overarching aim of the programme 'Stimulating evidence based, personalized and tailored information provision to improve decision making after oesophagogastric cancer diagnosis' (SOURCE) is to provide oesophagogastric cancer patients at all disease stages with evidence based and personalized information about survival, treatment-related side-effects and/or complications and HRQL, tailored to patients' specific information needs, to facilitate informed decision making about treatment and thereby optimize personal care and outcomes.

For this purpose, we designed the Source tool and training. The Source tool is a website to be used by care givers for informing patients about the outcomes of treatment. Prediction models are developed and built in the website in order to generate a personalized prediction of the outcomes: survival, toxicity and/or complications and HRQL. These predictions are visualized in clear and comprehensible graphs with a broad variation of options available for tailoring of the visualizations.

In order for care givers to be able to use this tool effectively, we designed the Source training. This communication skills training is comprised of an e-learning, two face-to-face group sessions and an individual booster session. Aside from an instruction video on the navigation within the Source tool, the e-learning consists of theory and tips and tricks on how to inform patients and communicate risks. The face-to-face components of the training are focused on getting the skilled use of the source tool into practice, by receiving personal feedback on the performance. Amongst the tackled problems are: how to tailor information to patients' needs, how to understandably communicate risk while not taking away patients' hope and how to use information in the context of a shared decision.

3. OBJECTIVES

We aim to test the effectiveness of the tool ('Source') and the communication skills training for health care providers.

3.1. The primary research question is:

Does the combination of the tool and the training improve observed (numerical) precision of information about treatment outcomes in clinical consultations?

3.2. The secondary research questions are:

Does the combination of tool and training affect:

- Health care providers' personalization of treatment outcome information
- Health care providers' tailoring of treatment outcome information
- Health care providers' use of visualizations
- Health care providers' use of time frame, initiative, treatment outcome category (on an utterance level)

- Health care providers' observed (numerical) precision of information about treatment outcomes in simulated patient consultations (effect training only)
- Health care providers' intention to inform patients about treatment outcomes using numbers (effect training only)
- Patient and physician satisfaction with communication and decision making
- Patient-reported shared decision making
- Patients' evaluation of the decision made
- Patients' evaluation and knowledge of the information provided by the health care provider
- Patients' health related quality of life
- Patients' trust in the health care provider
- Patients' anxiety
- Patients' helplessness/hopelessness
- Consultation time
- The (provisional) treatment decision made

Additionally we will assess:

- Demographical and personal variables
- Health care providers' use of the Source tool
- Health care providers' evaluation of the Source tool
- Health care providers' evaluation of the Source training + e-learning

4. STUDY DESIGN

4.1. Design

A multi-center pragmatic cluster stepped wedge design will be adopted. All health care providers will receive the intervention, i.e. the training and tool but at different time slots. These time slots are dependent on the geographical region (see Figure 1). Before the intervention takes place, a period of four to six months (taking the holiday season into account) will be used to include patients in order to collect control measurements. The subsequent transition period, in which the intervention will take place, will take approximately three to four months. After the intervention has taken place, again a period of four to six months is scheduled in which participating health care providers will include patients for measurements. Each participating health care provider (cluster) will include approximately three patients before and three patients after the intervention has taken place. The number of three patients per care provider is used as a mean, the minimum will be set at two patients and the maximum at six. The consultation with these patients will be audio recorded and providers and patients are asked to fill in short questionnaires before and after the consultation (see paragraph 7.2). In addition, each health care provider will do one simulated patient consultation before and one after training, which will also be recorded on audio.

4.2. Duration

Inclusion and data collection will take approximately 2 years. Figure 1 shows the planning of the study.

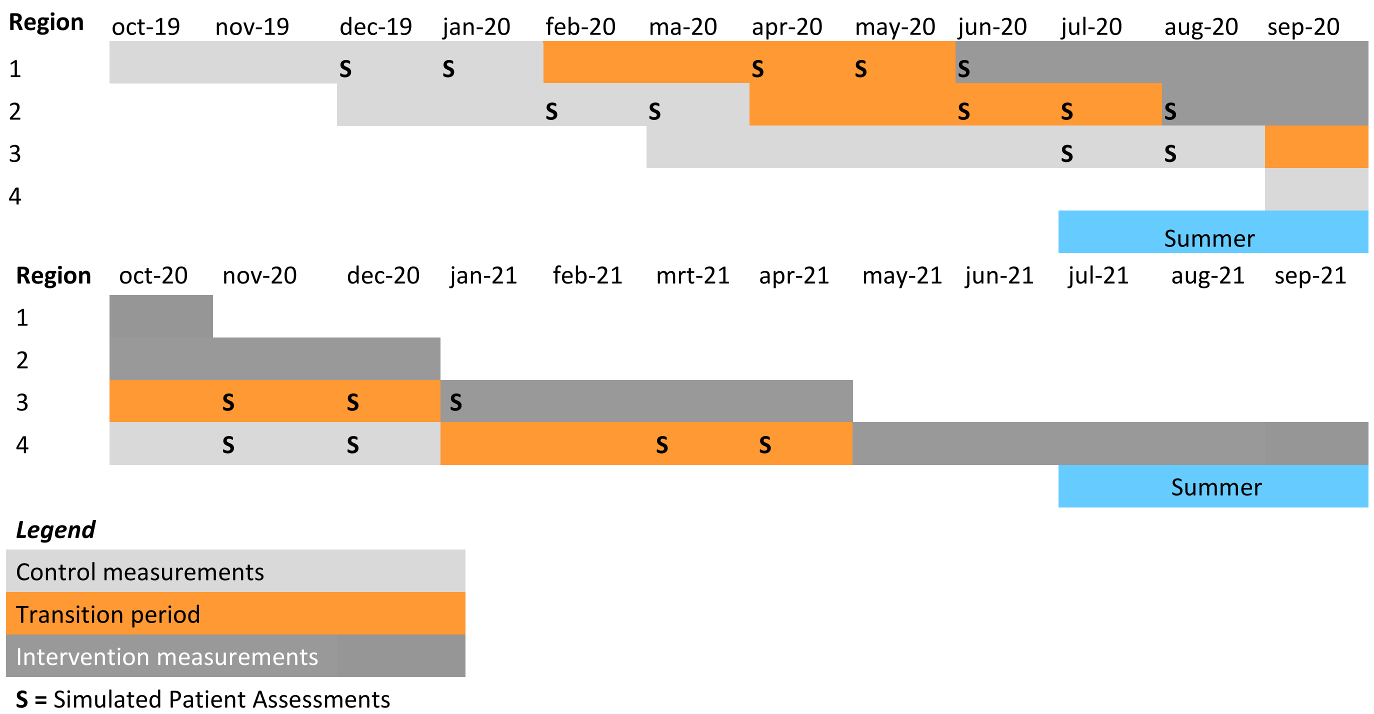

Figure 1. A schematic representation of the study planning over time

4.3. Setting

The study will be conducted at the medical oncology, radiotherapy and surgical departments of both academic and peripheral hospitals in the Netherlands.

4.4. Design over time

Figure 2 shows the design of the study over time for health care providers.

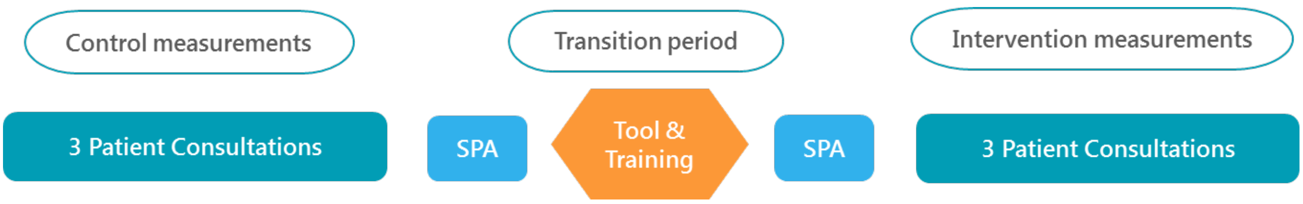

Figure 2. A schematic representation of the study design over time for health care providers, *SPA = Simulated Patient Assessment.*

5. STUDY POPULATION

5.1. Population (base)

All patients in participating centers, who have been diagnosed with oesophageal or gastric cancer and are scheduled for a treatment information consultation, will be asked to participate. The treatment information consultation is defined as the consultation in which one of the health care providers' main goals is to inform the patient on the outcomes of treatment(s), for example when decisions about treatment have to be made.

All oncologists, radiotherapists and surgeons (specialists and fellows), physician assistants and nurse practitioners conducting these consultations with oesophageal and gastric cancer patients, will be asked to participate.

5.2 Inclusion criteria

Health care providers:

- Specialist, physician assistant or nurse practitioner in the field of oncology, oncological radiotherapy or oncological surgery who are used to discussing treatment and treatment outcome with patients
- Informed consent for data collection
- Three audiorecorded treatment information consultations with oesophageal or gastric cancer patients before the scheduled transition period (control measurements)
- Three audiorecorded treatment information consultations with oesophageal or gastric cancer patients after the scheduled transition period (intervention measurements) and before the scheduled end date of the study

Patients:

- Age ≥ 18 years
- Histological or cytological proof of oesophageal or gastric cancer
- Informed consent for trial data collection

  A treatment information consultation with a participating doctor to discuss either curative treatment, first line palliative treatment, and/or best supportive care.

5.3 Exclusion criteria

Health care providers:

- Less than two control and/or intervention measurements

Patients:

- Cognitive impairment or insufficient understanding of the Dutch language
- GIST and smallcell tumors

5.4 Sample size calculation

The intervention is considered successful if a significant difference is observed in the precision of information about treatment outcomes provided by the health care providers (primary outcome) before and after the intervention. Assuming a medium sized effect (0.5 SD), an intracluster correlation of 0 and a power of 80%, 21 health care provider , i.e. clusters, are needed with 6 patients per health care provider (3 control and 3 post intervention). Thus, in total 21 health care providers and 126 patients need to be included in the study. Based on a predicted response rate of 50 % 252 patients will be approached.

6. TREATMENT OF SUBJECTS

6.1. The Source tool

The Source tool is designed for use in the consultation room. It aims to assist health care providers with giving evidence based information about treatment outcomes by providing visualizations of personalized predictions of treatment outcomes for different treatments of oesophageal and gastric cancer. These predictions result from recently designed and updated prediction models on the survival, toxicity, complications and health related quality of life of oesophageal and gastric cancer patients.

Data:
For survival models data from the nationwide Netherlands cancer registry were used. For the potentially curative and palliative settings, a multivariate Cox regression models were created and validated according to a temporal internal-external scheme (discriminative ability (c-indices): 0.71 for metastatic oesophagogastric cancer and 0.70 for metastatic gastric cancer, and 0.76 for the potentially curative models).[9]
For toxicity and complications of treatments, extensive meta-analysis data were used. For toxicity the literature was searched for prospective phase II or III randomized controlled trials (RCTs) on palliative first-line systemic treatment for advanced esophagogastric cancer. All CTCAE 1–2 (mild) and 3–4 (severe) adverse events from each chemotherapy arm were extracted and pooled by using single-arm meta-analysis.[10] For complications of treatment, databases were searched for randomized controlled trials investigating curative treatment regimens for gastric cancer. All grade 1–2 (mild) and grade 3–5 (severe) adverse events were extracted per cytotoxic regimen and combined in a single-arm random-effects meta-analysis. [11]
For health related quality of life, data from the national registry on health related quality of life of oesophageal and gastric cancer patients (POCOP) was used to create a mixed-effects model with treatment as a predictor based on 725 potentially curable patients. For metastatic patients, data from a meta-analysis on health related quality of life in the metastatic setting is provided.[12]
All models in the Source tool are updated when new, relevant data are available.

Visualizations:

- For survival data, both icon arrays and Kaplan Meier curves are implemented in order to be able to tailor the presentation format to individual patients. Adjustments to the displayed treatments and time period can be done, as well as to the visibility of confidence intervals. In the Kaplan Meier curve there is a possibility of showing three scenarios of survival[13,14] by shading the areas under the curve.
- For toxicity and complications (labeled as side effects) data, bar colored bar charts for both top 3 and all side effects are available. All treatments and side effects can be displayed separately or can be compared in one display.
- For health related quality of life data, simple line graphs are available for overall quality of life, symptom and functioning scores. Display of time period, confidence interval and general population (reference) scores can be adjusted.
- All visualizations can be joined in a simple overview of the three outcome categories (survival, side-effects, quality of life). An automatically generated text will accompany the graphs to serve as a summary of the communicated treatment outcomes. This overview can be saved as PDF or printed and handed to the patient.

6.2. The Source training

The training aims to teach participants to inform patients about outcomes of treatment. The training is placed in the context of shared decision making (SDM), following the 4-step model by Stiggelbout et al., 2015.[8]

The training is provided in small groups (3-6 participants) by an experienced trainer and supported by professional actors. The training aims to address knowledge, attitude and skills. The learning goals of the training are:

- *Knowledge:* After the training, the participant knows: (1) the basic theory of information giving (relevance, goal and tips and tricks), (2) the framework of SDM applied to both situations in which there is one preferred option and in which there are more conceivable options, (3) the do's and don'ts when giving risk and benefit information, (4) the functionalities of the Source tool and the data and calculations behind the Source tool.
- *Attitude:* After the training, the participant is aware of one's goal when informing patients about treatment and treatment outcomes and one's barriers to use evidence-based information about treatment outcomes. The participant has a generally positive attitude towards using evidence based information about treatment outcomes and is willing/motivated to use this information (by using the Source tool) in one's own consultations.
- *Skills:* After the training, the participant is capable of applying the required (risk) information giving skills in consultations about treatment and treatment outcomes and is able to use evidence-based information when doing so. These two learning goals include: (1) giving evidence-based information in a manner which patients can understand, while not taking away all their hopes, (2) tailoring the amount and content of the information to individual patients' needs, (3) using information giving skills to facilitate patients decision making.

The training includes:

1. Access to an e-learning providing theory and tips and tricks
2. Two half-day training sessions with instruction, role play and personal feedback
3. An individual (or pairwise) booster session of 1 hour
4. Access to an online consultation room tool, providing evidence-based information on outcomes of treatment for oesophageal and gastric cancer patients (the Source tool, see 6.1)

7. METHODS

7.1. Study parameters/endpoints

7.1.1. Main study parameters/endpoints

The primary outcome is (numerical) precision of information about outcomes of treatment in the treatment information consultation. To observe this, we will audiotape and content code the consultation, using the SOURCE observational scale, to assess the primary outcome.

7.1.2. Consultation - secondary study parameters/endpoints

- Health care providers' personalization of treatment outcome information (SOURCE observational scale)
- Health care providers' tailoring of treatment outcome information to patients' information need (SOURCE observational scale)
- Health care providers' use of visualizations (SOURCE observational scale)
- Utterance level: health care providers' use of time frame, initiative for discussing outcomes (health care provider, patient, informal caregiver), treatment outcome category discussed (survival, side-effects, quality of life) (SOURCE observational scale)
- Consultation time

7.1.3. Health care provider - secondary study parameters/endpoints

- Health care providers' satisfaction with communication and decision making (Patient Satisfaction Questionnaire (PSQ-5 doctor version))
- Health care providers' use of the Source tool (observation)
- Health care providers' evaluation of the Source tool (tailor made evaluation questionnaire)
- Health care providers' evaluation of the Source e-learning (tailor made evaluation questionnaire)
- Health care providers' evaluation of the Source face-to-face training (tailor made evaluation questionnaire)
- Health care providers' intention to inform patients about treatment outcomes using numbers (effect training only) (CPD reaction questionnaire)

7.1.4. Patient – secondary study parameters/endpoints

- Patient satisfaction with communication and decision making (Patient Satisfaction Questionnaire (PSQ-5))
- Patients' evaluation and knowledge of the information provided by the health care provider (INFO-25 + tailor made items on information about outcomes of treatment)
- Patient-reported shared decision making (Shared Decision Making Questionnaire (SDMQ-9))
- Patients' evaluation of the decision made (Decisional Conflict Scale (DCS))
- Patients' health related quality of life (EORTC QLQ-C30 + EORTC QLQ-OG25)
- Patients' trust in the health care provider (Trust in the Oncologist Scale (TiOS-sf))
- Patients' anxiety (State-Trait Anxiety Inventory (STAI))
- Patients' helplessness/hopelessness + fighting spirit ((Mini-)Mental Adjustment to Cancer scale ((Mini-)MAC))
- Patients' (provisional) treatment decision (medical record)

7.1.5. Demographical and personal study parameters

Health care providers:

- Age
- Gender

- Nationality
- Level of medical training/ job position
- Specialism
- Earlier communication skills training

Patients:

- Tumour location
- Age
- Gender
- Personal living situation
- Number of children (under 18)
- Nationality
- Educational level
- Perceived efficacy in communication before the consultation
- Information need
- Experience with graphs/numbers

7.2. Study procedures

7.2.1. Patient study procedures

Patients with oesophageal or gastric cancer who are scheduled for a treatment information consultation, will be approached by telephone for informed consent by a SOURCE research employee. Informed consent forms together with the information letter and the baseline T0 questionnaire will be sent to the patient by mail or email.
Consenting patients will be asked to fill in a questionnaire at home before the start of the consultation (T0 P; 7-15 minutes) and one in the waiting room, just before the start of the consultation (T1 P; 3-5 minutes).
Together with receiving T1, patients will receive an envelope containing the T2 questionnaire (10-20 minutes). Patients will be asked to fill in the T2 questionnaire within a time period of two weeks after the consultation. The treatment information consultation will be audio recorded. Dependent on the procedures as agreed upon with the participating centers, either the research nurse of the participating center or a researcher of the SOURCE study will facilitate the audio-recording and questionnaire completion.
Three months after the consultation, patients will be asked to fill in the last questionnaire (T3 P; 10-15 minutes). Patients participating in the POCOP trial will not receive this questionnaire. For these patients, the data from the T2 questionnaire will be acquired through the POCOP database.

7.2.2. Health care provider study procedures

Patient inclusion

After informed consent is signed by the health care provider, each consultation with a participating patient will be recorded on audio. After the consultation, health care providers will fill in a short questionnaire (T2 HCP; 1-2 minutes) on their evaluation of the consultation. Health care providers’ use of Source tool, will be logged. Each action will be logged and labeled by

moment in time. Entered patient data and health care providers' study code will be stored using 512-bit RSA encryption.

Simulated patient assessment 1 (before intervention)

During the control measurement period (see Figure 2), the first simulated patient assessment (SPA) will take place, which will be recorded on audio. At the first SPA, health care providers will be asked to fill in T1 HCP (3-5 minutes). After the consultation, health care providers will also fill in T2 HCP.

Transition period

After completion of the e-learning, health care providers will be asked to fill in a short evaluation questionnaire on the e-learning (T3 HCP; 5-10 minutes).

Simulated patient assessment 2 (after intervention)

After training is completed, the second SPA will take place. At the second simulated patient assessment, health care providers will fill in T4 HCP (5-10 minutes) and T2 HCP.

End of study

Lastly, at completion of their patient inclusion, health care providers will fill in a short evaluation of the tool (T5 HCP; 5-10 minutes).

7.3. Withdrawal of individual subjects

Subjects can leave the study at any time for any reason if they wish to do so without any consequences. The investigator can decide to withdraw a subject from the study for urgent medical reasons.

7.4. Replacement of individual subjects after withdrawal

Participating patients will only be replaced after withdrawal if the minimum of 126 audio recoded consultations in total or the minimum of 2 control measurements and 2 interventions measurements per health care provider is not reached.

Participating health care providers that did not succeed in including 2 control measurements and 2 interventions measurements will only be replaced if the minimum of 21 in total is not reached. Health care providers who withdraw during the study will only be replaced if they drop out after informed consent, but before training.

7.5. Premature termination of the study

Not applicable, as we do not expect the study to be terminated prematurely due to proven un-safety.

8. SAFETY REPORTING

The investigator will inform the subjects if anything occurs, on the basis of which it appears that the disadvantages of participation may be significantly greater than was foreseen in the research

proposal. Safety of the participants will be reconsidered and if considered insufficient, the study will be suspended. The investigator will take care that all subjects are kept informed.

9. STATISTICAL ANALYSIS

9.1. Primary study parameter

Audio tapes of consultations will be analyzed, using Noldus' The Observer XT software, on the primary outcome. Time-based analysis on all utterances of information on treatment outcomes is performed. Each utterance will receive a code based on the study specific coding scheme.
Utterance scores will be summarized on the level of the consultation and compared between control and intervention measurements in a within-subjects analysis. Effects with one-sided p-values < .05 will be considered significant; 95CI's as well as effect sizes will be provided.
Baseline differences across the compared patient groups on socio-demographic (age, gender, educational level, etc.) and clinical characteristics (cancer phase at time of the consultation, time since diagnosis, etc.) as well as patients' baseline measures will be examined with the appropriate statistical tests (ANOVA, Chi-squared tests). All analyses of effectiveness will be controlled for specialization of the health care provider, profession of the health care provider (specialist, fellow, nurse specialist, physician assistant) and center specific characteristics (academical or peripheral hospital, starting time of intervention).
Reasons for missing data will be registered and recorded. Dependent on the type of missing data, the appropriate procedures will be used. If necessary, multiple imputation could be applied.

9.2. Secondary study parameters

The secondary outcomes based on the consultation, both on the level of the utterance or the level of the consultation) will be coded likewise by the study specific coding scheme. Effects of the intervention on the outcomes will be analyzed in the same manner as the effect on the primary outcome (see 9.1).
The effect on the secondary outcomes measured by patient or health care provider self-report will be analyzed in multi-level regression analysis.
The effect of the training on the observed (numerical) precision of information about outcomes of treatment in simulated patient encounters will be assessed by using a repeated measure ANOVA.
To answer questions regarding the evaluation of the intervention (tool and training), the appropriate descriptive statistics will be used (medians, percentages).

10. ETHICAL CONSIDERATIONS

10.1. Recruitment and consent

All procedures, including the interventions and questionnaires, were tested in a pilot study (2019).

10.1.1. Health care providers

Health care providers will be informed about the study and asked for consent by the investigator, both face-to-face and on paper. All health care providers will be invited to participate in a

standardized patient assessment, an e-learning, two training half-days, one booster session and again one standardized patient assessment. Accreditation for this training is available (14 accreditation points).

10.1.2. Booster recordings + booster sessions

For the booster session, as part of the training, an audio recording of a simulated consultation will be used to provide the health care providers with personalized feedback. Each of the health care providers in the training condition will participate in one audio-recorded simulated patient consultation. In the individual booster session with the trainer, the health care provider will recieve feedback on their performance in the consultation.

10.1.3. Patients in the trial

1. *Invitation.*

Eligible patients will be identified from the outpatient clinic agendas of the participating health care providers by either the health care provider, a research nurse or a research associate (depending on the logistical agreements with the individual center). If possible, the treating health care provider will be asked for permission to contact the patient. Since the role of treating health care provider is not in every case divided before consultation, this cannot always be realized.
Either the research nurse of the research associate will contact the patient by telephone, explain the study and invite to participate. We strive to allow patients at least 3 days' time to consider their participation in the study and therefore preferably contact the patient as soon as the consultation is planned. Via telephone, patients will be informed about the nature and goal of the study. Patients who consent, will be contacted by post. All subsequent (postal) contact will be arranged by the coordinating investigators in the Amsterdam UMC, location AMC.

2. *Informed consent and baseline questionnaire*.

After oral consent, patients will receive a written information letter with information about the study, informed consent forms and the baseline questionnaire (T0 P) to be filled in at home, accompanied by a return envelope. Since time between initial contact and consultation is often short, patients will not in every case be able to fill in the informed consent forms and the baseline questionnaire before consultation. Patients will still receive a written information letter with information about the study, informed consent forms and T0 P but will in this case be asked to fill these in when meeting the research associate in the waiting room for T1 P. By signing informed consent patients and their family agree to the collection of audio recording and questionnaire data.

3. *Waiting room.*

A research associate or research nurse will meet the patient (and their family) in the waiting room, and check for the filling in of informed consent forms as well as the baseline questionnaire. Patients will again be given the opportunity to ask questions. Patients will be informed about the procedure of recording the consultation (and the small audio recorder will be

shown). The associate or nurse will check whether the family members present have provided consent for audio recording the consultation. The patient will be asked to fill out a two page questionnaire (T1 P). In addition, the patient will receive the post-consultation questionnaire (T2 P) with a return envelope.

4. *Audio recording*.
Prior to the outpatient clinic appointment, the health care provider will be informed about the fact that the patient is participating in SOURCE and that the consultation will be audio-recorded. Either the research associate, the research nurse or the health care provider will operate the recorder (depending on local agreements). If done by a research nurse or associate, they won't be present during the consultation. Patient and family won't be approached after the consultation. Encrypted audio devices will be used as soon as available to prevent access after the recording has been saved on the device. All audio recordings will as soon after the consultation as possible be filed in a strict protective digital repository.

5. *Follow up*.
Patients will receive one more follow-up questionnaires at 3 months post- consultation (T3 P). Their medical files will be checked for information about treatment until 12 months post-consultation, either by a research associate or a research nurse.

10.2. Compensation for injury

Not applicable, as this is a non-WMO study and participating in the study is without risks. We estimate the physical burden/risk to be none. We are aware of a potential emotional/psychological burden for participants. Yet, we estimate the chance of any persistent harmful effect to be negligible. We have taken care to minimize the confrontational nature of the study material. We have conducted a pilot study on the study procedures, the interventions and the questionnaires. Moreover, potential participants will be informed about the topic of the study, i.e. doctor-patient communication. Participants can voluntarily decide if they feel up to participation, and they can leave the study at any time for any reason if they wish to do so without any consequences. In case of psychological distress, participants will be referred to the locally available support. Also, the investigator can decide to withdraw a subject from the study if he/she thinks that is in the best interest of the participant. Based on this argumentation, we estimate the risk of participation to be negligible.

11. ADMINASTRIVE ASPECTS AND PUBLICATION

11.1. Handling and storage of data and documents

The investigator ensures that the handling of personal data complies with the Dutch Personal Data Protection Act. Patients will be assigned a unique code which will be used on all documents, and which does not contain any personal information such as date of birth or names. The investigator holds the key to the code in a locked file stored separately from data. Data will be stored for 15 years.

11.2. Amendments

Amendments are changes made to the research after a favorable opinion by the accredited METC has been given. All amendments will be notified to the METC that gave a favorable opinion.

11.3. Public disclosure and publication policy

Investigators are entitled to disseminate the findings of the study via publications in reputable scientific journals and via presentations at seminars or scientific conferences. The investigators carry final responsibility for the scientific content of the publication on the main findings of the study. No limitations to the disclosure and publication of the findings have been imposed by the sponsor. The trial is registered in a public trial registry (clinicaltrials.gov) under *NCT04232735,* https://clinicaltrials.gov/ct2/show/NCT04232735. From each of the eleven participating centers, every fully educated health care provider is permitted to join the publication of results as a co-author under the following conditions:

- The health care provider has participated in two simulated patient assessments.
- The health care provider has completed the entire training program.
- The health care provider has provided three consultation recordings before and after the intervention.
- The health care provider carefully reads and comments on the provided manuscript on the results of the study.

Disclosure: the SOURCE study is endorsed by the Dutch Upper GI Cancer Group (DUCG) and funded by the Dutch Cancer Society (KWF Kankerbestrijding).

12. REFERENCES

1. Jacobs M, Macefield RC, Elbers RG, Sitnikova K, Korfage IJ, Smets EM, Henselmans I, van Berge Henegouwen MI, de Haes JC, Blazeby JM, Sprangers MA. Meta-analysis shows clinically relevant and long-lasting deterioration in health-related quality of life after esophageal cancer surgery. Qual Life Res 2014;23:1155-1176.
2. Al-Batran SE, Ajani JA. Impact of chemotherapy on quality of life in patients with metastatic esophagogastric cancer. Cancer 2010;116:2511-2518.
3. Thrumurthy SG, Morris JJ, Mughal MM, Ward JB. Discrete-choice preference comparison between patients and doctors for the surgical management of oesophagogastric cancer. Br J Surg 2011;98:1124-1131.
4. Hitz F, Ribi K, Li Q, Klingbiel D, Cerny T, Koeberle D. Predictors of satisfaction with treatment decision, decision-making preferences, and main treatment goals in patients with advanced cancer. Support Care Cancer 2013;21:3085-3093.
5. Jacobs M, Macefield RC, Blazeby JM, Korfage IJ, van Berge Henegouwen MI, de Haes HC, Smets EM, Sprangers MA. Systematic review reveals limitations of studies evaluating health-related quality of life after potentially curative treatment for esophageal cancer. Qual Life Res 2013;22:1787-1803.
6. Takeuchi EE, Keding A, Awad N, Hofmann U, Campbell LJ, Selby PJ, Brown JM, Velikova G. Impact of patient-reported outcomes in oncology: a longitudinal analysis of patient-physician communication. J Clin Oncol 2011;29:2910-2917.

7. McNair AG, Brookes ST, Davis CR, Argyropoulos M, Blazeby JM. Communicating the results of randomized clinical trials: do patients understand multidimensional patient-reported outcomes? J Clin Oncol 2010;28:738-743.
8. Stiggelbout, A. M., Pieterse, A. H., & De Haes, J. C. (2015). Shared decision making: Concepts, evidence, and practice. Patient Educ Couns, 98(10), 1172-1179. doi: 10.1016/j.pec.2015.06.022
9. van den Boorn, H. G., Abu-Hanna, A., Ter Veer, E., van Kleef, J. J., Lordick, F., Stahl, M., ... & Bang, Y. J. (2019). SOURCE: A registry-based prediction model for overall survival in patients with metastatic oesophagogastric or gastric cancer. Cancers, 11(2), 187.
10. Lok Lam Ngai, Emil ter Veer, Héctor G. van den Boorn, E. Hugo van Herk, Jessy Joy van Kleef, Martijn G. H. van Oijen & Hanneke W. M. van Laarhoven (2019) TOXview: a novel graphical presentation of cancer treatment toxicity profiles, Acta Oncologica, 58:8, 1138-1148, DOI: 10.1080/0284186X.2019.1601256
11. Tom van den Ende, Frank A. Abe Nijenhuis, Héctor G. van den Boorn, Emil ter Veer, Maarten C. C. M. Hulshof, Suzanne S. Gisbertz, Martijn G. H. van Oijen, Hanneke W. M. van Laarhoven. (2019) COMplot, A Graphical Presentation of Complication Profiles and Adverse Effects for the Curative Treatment of Gastric Cancer: A Systematic Review and Meta-Analysis. Frontiers in Oncology 9.
12. van Kleef, J. J., ter Veer, E., van den Boorn, H. G., Schokker, S., Ngai, L. L., Prins, M. J., ... & Sprangers, M. A. (2019). Quality of Life During Palliative Systemic Therapy for Esophagogastric Cancer: Systematic Review and Meta-Analysis. JNCI: Journal of the National Cancer Institute.
13. Kiely, B. E., Soon, Y. Y., Tattersall, M. H., & Stockler, M. R. (2010). How long have I got? Estimating typical, best-case, and worst-case scenarios for patients starting first-line chemotherapy for metastatic breast cancer: a systematic review of recent randomized trials. Journal of Clinical Oncology, 29(4), 456-463.
14. Kiely, B. E., Stockler, M. R., & Tattersall, M. H. (2011). Thinking and talking about life expectancy in incurable cancer. In Seminars in oncology (Vol. 38, No. 3, pp. 380-385). WB Saunders.
